# Surface Engineering of Electrospun PLA Fibers via Chitosan/Hyaluronic Acid Polyelectrolyte Complexes for Tunable Release of Rosmarinic Acid

**DOI:** 10.3390/polym18101207

**Published:** 2026-05-15

**Authors:** Selin Kyuchyuk, Dilyana Paneva, Milena Ignatova, Nevena Manolova, Iliya Rashkov, Daniela Karashanova, Milena Mourdjeva, Nadya Markova

**Affiliations:** 1 Laboratory of Bioactive Polymers, Institute of Polymers, Bulgarian Academy of Sciences, Akad. G. Bonchev St, Bl. 103A, BG-1113 Sofia, Bulgaria; selin.erdinch@polymer.bas.bg (S.K.); panevad@polymer.bas.bg (D.P.); manolova@polymer.bas.bg (N.M.);; 2 Institute of Optical Materials and Technologies, Bulgarian Academy of Sciences, Akad. G. Bonchev St, Bl. 109, BG-1113 Sofia, Bulgaria; dkarashanova@yahoo.com; 3 Institute of Biology and Immunology of Reproduction “Akad. Kiril Bratanov”, Bulgarian Academy of Sciences, 73, Tsarigradsko Shose Blvd., BG-1113 Sofia, Bulgaria; mourdjeva@ibir.bas.bg; 4 Institute of Microbiology, Bulgarian Academy of Sciences, Akad. G. Bonchev St, Bl. 26, BG-1113 Sofia, Bulgaria; nadya.markova@microbio.bas.bg

**Keywords:** electrospinning, poly(L-lactide), coating, polyelectrolyte complex, chitosan, hyaluronic acid, rosmarinic acid, antioxidant activity, antimicrobial properties

## Abstract

In this study, a hierarchical design strategy is introduced for tuning the release of rosmarinic acid (RA) from electrospun poly(L-lactide) (PLA) fibrous materials via surface engineering with chitosan/hyaluronic acid (Ch/HA) polyelectrolyte complexes (PECs). RA was selectively incorporated within the fiber bulk, the PEC coating, or both, enabling control over its spatial distribution. The PEC coating, formed by sequential dip coating, was shown to act as a diffusion-regulating layer with a dual role—either retarding RA release or promoting rapid initial release when functioning as a surface-associated reservoir. As a result, the release kinetics could be systematically tuned depending on the coating architecture and RA localization. Thorough characterization confirmed successful coating formation, enhanced surface hydrophilicity, and improved mechanical performance. All RA-loaded materials retained high antioxidant activity and exhibited pronounced antibacterial and antifungal effects against *Staphylococcus aureus*, *Escherichia coli*, and *Candida albicans*. This work introduces PEC-modified electrospun systems as a versatile platform for the rational design of multifunctional fibrous biomaterials with controlled release profiles, with potential applications in wound healing and drug delivery.

## 1. Introduction

Electrospinning is considered as a versatile and highly efficient technique for the one-step fabrication of micro- and nanoscale fibers from a wide range of polymers and polymer blends by applying a high voltage to their solutions or melts [1,2,3,4,5,6,7]. This process has gained significant attention owing to its numerous advantages compared to other methods for the preparation of polymer fibers, namely: the simplicity of the required equipment, the feasibility of producing lightweight fibrous materials with high surface area, and the ability to incorporate a wide variety of bioactive natural or synthetic low-molecular-weight agents. The combination of electrospinning with post-fabrication modification of the fibers enables tuning of the physicochemical behavior of the fibrous materials, like hydrophilic/hydrophobic balance and mechanical properties; as well as, e.g., imparting bioactivity to biologically inert fibrous materials [8]. The preparation of a functional polymer coating via dip coating is a facile and effective approach for modification of the surface of electrospun materials [8,9,10].

The electrospinning of poly(L-lactide) (PLA) is widely applied for the preparation of fibrous materials [11,12], and this is related to the advantages of PLA, including its renewable origin, biocompatibility, biodegradability, favorable mechanical properties, and its facile electrospinning from a solution. However, PLA lacks inherent bioactivity, and pathogenic microorganisms readily adhere to its surface, thus promoting the formation of harmful bacterial biofilms [9,13]. This lowers the applicability of PLA-based materials in the biomedical practice. A straightforward approach for imparting antimicrobial behavior to electrospun PLA-based materials is the preparation of a chitosan (Ch) coating by a dip coating technique using aqueous acidic Ch solution [9,14]. Ch has inherent antimicrobial activity [15,16,17] and is a suitable partner of PLA. Ch is soluble in aqueous acidic medium; thus, water-insolubility has to be imparted at a subsequent stage to its coating on the fibers. Owing to the ionizable in aqueous acidic medium amino groups in the structure of Ch, this polysaccharide as a polycation has the ability to form water-insoluble polyelectrolyte complexes (PECs) with oppositely charged natural or synthetic polyanions [18,19,20]. Hyaluronic acid (HA) is a typical example of a natural polyanion that can ionically interact with Ch, thus forming a water-insoluble PEC [21,22]. Hence, the combination of electrospinning and dip coating and subsequent formation of PEC between Ch and HA might lead to the preparation of fibers with a coating that is stable in aqueous medium. Furthermore, the incorporation of HA in fibrous materials with potential medical application is of interest since this anionic polysaccharide, which is a component of the extracellular matrix, is highly biocompatible and non-toxic and shows antioxidant, anti-inflammatory, wound healing and anticancer activity [23,24]. So far there are no data on the preparation of PLA fibers coated with PEC based on Ch and HA pair. RA is a naturally occurring polyphenolic compound having antioxidant and antimicrobial activity [25,26]. Therefore, it is a promising bioactive component of electrospun fibrous materials with potential biomedical applications. It has been demonstrated that RA loading in polymer fibers by electrospinning of blend solutions does not affect its valuable biological behavior [27,28,29,30,31]. No data have been found on the preparation of PLA fibrous material coated with PEC between Ch and HA with RA loaded in PLA fibers and/or in PEC coating.

The aim of the present study is to develop and systematically investigate electrospun PLA-based fibrous materials with tunable release of rosmarinic acid via surface engineering using chitosan/hyaluronic acid polyelectrolyte complexes. The working hypothesis is that the architecture of the PEC coating and the spatial distribution of RA within the composite system will govern the release kinetics and, consequently, the antioxidant and the antimicrobial performance of the materials.

To test this hypothesis, PLA and PLA/RA fibrous materials were fabricated by electrospinning and subsequently modified by sequential dip coating to form a PEC layer with a controlled composition and deposition sequence. The resulting materials were characterized in terms of morphology, surface chemistry, wettability, thermal and mechanical properties. Furthermore, the influence of material design on RA release behavior, antioxidant activity, and antimicrobial performance against representative bacterial and fungal strains was evaluated, with the objective of establishing structure–property–function relationships relevant to biomedical applications such as wound healing and drug delivery.

## 2. Materials and Methods

### 2.1. Materials

Poly(L-lactide) (PLA) (Ingeo™ Biopolymer 4032D, Nature Works LLC, Plymouth, MN, USA; M_w_ = 259,000 g/mol; M_w_/M_n_ = 1.94; as estimated by size-exclusion chromatography using polystyrene standards) was used. Chitosan (Ch) with a deacetylation degree of 75–85% and Mη of 340,000 g/mol was purchased from Aldrich, St. Louis, MO, USA, and sodium hyaluronate (HA) from Acros Organics, Geel, Belgium. Rosmarinic acid (RA) (Merck, Billerica, MA, USA), Rhodamine B (RhB) and 2,2-diphenyl-1-picrylhydrazyl free radical (DPPH^•^) (Sigma-Aldrich, Darmstadt, Germany) were used as received. Dichloromethane (DCM) (Fisher Scientific UK, Loughborough, UK), glacial acetic acid (Merck, Billerica, MA, USA), dimethylformamide (DMF, Fisher Scientific UK, Loughborough, UK) and absolute ethanol (Merck, Billerica, MA, USA) were of analytical grade. The salts used to prepare the pH 7.4 buffer solution (KH_2_PO_4_ and Na_2_HPO_4_) were obtained from Merck Chemicals (Merck, Billerica, MA, USA). *Staphylococcus aureus* (*S. aureus*) 3359, *Escherichia coli* (*E. coli*) 3397, and *Candida albicans* (*C. albicans*) 74 were acquired from the National Bank for Industrial Microorganisms and Cell Cultures (NBIMCC), Sofia, Bulgaria.

### 2.2. Preparation of the Fibrous Materials from PLA and PLA/RA by Electrospinning

PLA mats were prepared by electrospinning of its solution in DCM/DMF 75/25 (*v*/*v*) at a polymer concentration of 10 *w*/*v*%. For PLA/RA mats, the RA content was 5 or 10 wt.% in respect to the polymer weight. For example, for the preparation of PLA/RA mats with RA content of 10 wt.% with respect to the polymer weight, a blend solution was obtained by mixing PLA (3.0 g) solution in 22.5 mL DCM and RA (0.3 g) solution in 7.5 mL DMF. The electrospinning was performed under the following conditions: constant feed rate of 1.5 mL/h obtained by using an infusion pump (NE-300 Just InfusionTM Syringe Pump, New Era Pump Systems Inc., Farmingdale, NY, USA), applied voltage of 20 kV (high-voltage power supply for scientific use, Model: HVG-CONT-LCD, Linari Engineering [Pisa, Italy]), tip-to-collector distance of 18 cm, deposition time of 28 h, use of a grounded rotating aluminum drum collector with rotation speed maintained at 1300 rpm, room temperature of 23 °C, and a relative humidity of 49%. The obtained fibrous materials were dried under reduced pressure at 45 °C for 24 h.

### 2.3. Preparation of Fibrous Mats from (Ch/HA)-coat-PLA, (Ch/HA)-coat-PLA/RA, [(Ch/RA)/HA]-coat-PLA, [(Ch/RA)/HA]-coat-PLA/RA, (HA/Ch)-coat-PLA, (HA/Ch)-coat-PLA/RA, [HA/(Ch/RA)]-coat-PLA and [HA/(Ch/RA)]-coat-PLA/RA

**Scheme 1:**
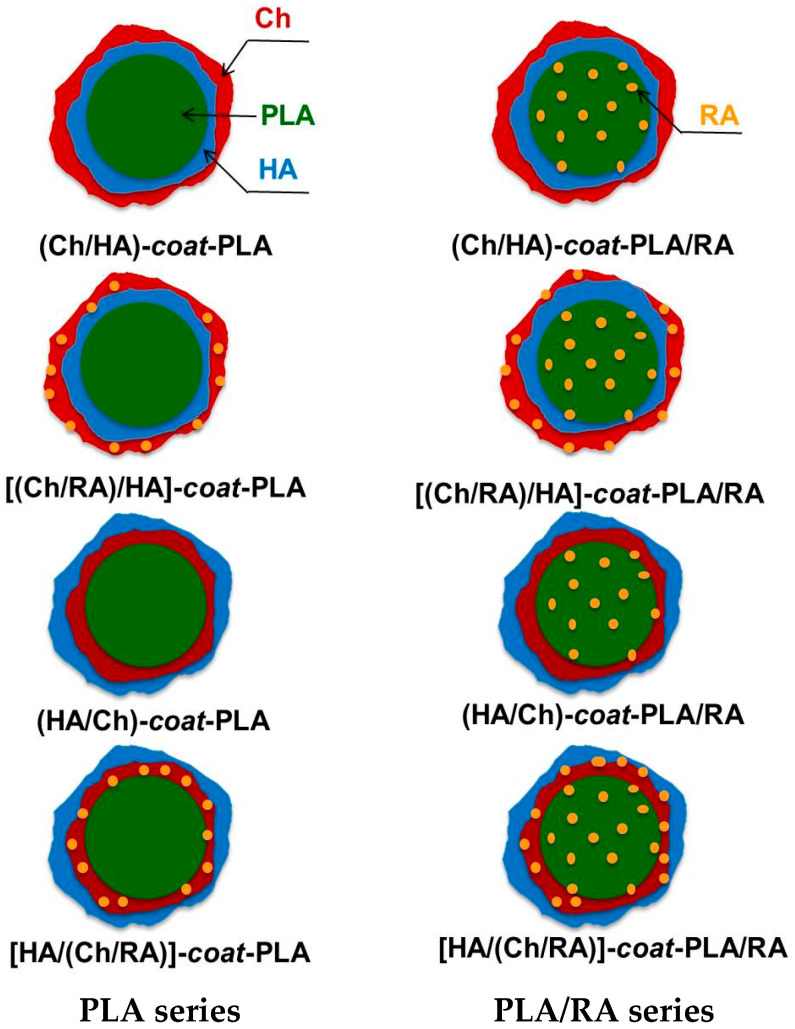
Schematic representation of the cross-section of a fiber prepared by electrospinning, dip coating and subsequent polyelectrolyte complex (PEC) formation.

A series of polyelectrolyte complex (PEC)-coated PLA or PLA/RA fibrous materials were prepared by deposition of a layer from one polyelectrolyte (Ch or HA) followed by deposition of a layer from the oppositely charged polyelectrolyte (HA or Ch) by applying dip coating using aqueous solution of the respective polyelectrolyte. Ch solution in the absence/presence of RA was used. Here, an example of the abbreviations used is provided: a PLA mat sequentially immersed in HA and Ch solution is denoted by (Ch/HA)-*coat*-PLA mat. The abbreviations assigned to the prepared PEC-coated fibrous materials are presented in Scheme 1. Ch solution prepared by dispersion of the polycation in distilled water and addition of drops of glacial acetic acid up to pH 4.5 was used. For preparation of Ch/RA solution at weight ratio of 1/1, a solution of RA (0.04 g) in abs. ethanol (12 mL) was added dropwise to 0.14% Ch (0.04 g) solution in acidic aqueous solution (pH 4.5, 28 mL). A 0.1% (*w*/*v*) HA solution in distilled water was used. For example, for the preparation of PLA fibrous materials coated with PECs (Ch/RA)/HA to obtain [(Ch/RA)/HA]-*coat*-PLA, the procedure was as follows: PLA mats were immersed in HA solution, left in the solution for 1 min, then withdrawn and dried at 25 °C for 1 day. After that the mats were immersed in Ch/RA solution for 1 min and dried at 25 °C for 2 days up to constant weight.

### 2.4. Characterization of the Fibrous Materials

The dynamic viscosity of the solutions was measured using a Brookfield DV-II+ Pro programmable viscometer (Middleboro, MA, USA) in a cone-and-plate configuration, including a cone spindle and a temperature-controlled sample cup maintained at 25 ± 0.1 °C. The conductivity of the spinning solutions was determined using an electrolytic cell with two rectangular platinum electrodes (surface area 0.6 cm^2^), following a previously reported method [32]. The cell was calibrated using standard KCl solutions prior to measurements.

The morphology of the (non)coated fibrous materials was examined by scanning electron microscopy (SEM) with a JSM-5510 (JEOL, Tokyo, Japan) microscope. Before imaging with a microscope, the samples were vacuum-coated with gold. The average fiber diameter and corresponding standard deviation were measured using ImageJ software (version 1.53e, Wayne Rasband, National Institutes of Health, Bethesda, MD, USA). Measurements were taken from at least 40 fibers selected from two separate SEM micrographs for each sample. Transmission electron microscopy (TEM) analysis was conducted using a JEM 2100 instrument (JEOL Ltd., Tokyo, Japan) operating at an accelerating voltage of 200 kV.

The presence of Ch or HA in the PEC coating on the fibers was visualized by Leica TCS SPE confocal laser scanning microscope (CLSM, Wetzlar, Germany). CLSM was equipped with a Leica Application Suite X (LAS X) Microscope Software, Version 3.5.7.23225 (Leica Microsystems CMS GmbH, Wetzlar, Germany). The visualization was achieved by using a layer from Ch/RA and from HA/RhB on PLA or PLA/RA fibers (RA and RhB were used as fluorescent markers). For that purpose, mats from [(Ch/RA)/(HA/RhB)]-*coat*-PLA and [(Ch/RA)/(HA/RhB)]-*coat*-PLA/RA, as well as mats from [(HA/RhB)/(Ch/RA)]-coat-PLA and [(HA/RhB)/(Ch/RA)]-*coat*-PLA/RA, were prepared. Ch/RA coating was prepared as described in Section 2.3. For preparation of HA/RhB coating, 0.1% (*w*/*v*) solution of HA in distilled water was used, in which RhB dye was used in a quantity necessary for the HA/RhB weight ratio to be 1/1. Fibrous samples with dimensions of 10 × 10 mm^2^ were deposited onto a glass slide. Two drops of immersion oil (Sigma-Aldrich, St. Louis, MO, USA) were applied to the sample surface and allowed to spread until complete wetting was achieved. The wetted samples were then covered with a cover glass. The immersion oil, with a refractive index of 1.516, was used to enable high-magnification imaging. All images were acquired using an ACS APO 63× oil immersion objective, with a drop of immersion oil placed between the cover glass and the objective, and a digital zoom factor of 3×. RA- and RhB-loaded materials were excited using 405 nm and 532 nm lasers, respectively. Fluorescence emission was collected in the ranges of 420–467 nm and 561–590 nm for the first and second channels, respectively. Two detection channels were used to sequentially record fluorescence signals from both lasers for each Z-layer. The fluorescence of RA- and RhB-loaded mats was recorded using a Leica DM5500Q confocal spectral detector TCS SPE L (Wetzlar, Germany). The acquired images had a pixel resolution of 2048 × 2048, corresponding to an image size of 58.2 × 58.2 μm^2^ and a digital resolution of 0.03 μm/pixel. The scanning was performed from the top layer of each electrospun fibrous sample to a depth of 15 μm using a Z-step of 1 μm. The total laser exposure was 8.4 min.

Attenuated total reflection-Fourier transform infrared (ATR-FTIR) spectra of the fibrous materials were registered by Nicolet™ iS™50 spectrometer (Thermo Fisher Scientific Inc., Waltham, MA, USA). An IR diamond iS50 ATR accessory (Thermo Fisher Scientific Inc., Waltham, MA, USA) was used with the spectrometer.

The surface chemical composition of the fibrous materials was analyzed using X-ray photoelectron spectroscopy (XPS). XPS measurements were carried out using an ESCALAB-MkII (VG Scientific, East Grinstead, UK) electron spectrometer equipped with an ultrahigh-vacuum (UHV) chamber. Spectra were recorded with a Mg Kα X-ray source. High-resolution spectra were energy-calibrated by referencing the C_1s_ peak at 285 eV. Data analysis was performed using CasaXPS software (version 2.3.25PR1-0). Peak fitting employed Gaussian–Lorentzian line shapes following subtraction of a Shirley background.

Static water contact angles (WCAs) were determined using an Easy Drop shape analysis system (DSA20E, Krüss GmbH, Hamburg, Germany). A computer-controlled dosing system was used to dispense a 10 μL water droplet onto the fiber surfaces. For each sample, 20 measurements were carried out.

The thermal stability of the fibrous materials was determined by thermogravimetry using a Perkin Elmer TGA 4000 Thermogravimetric Analyzer (Waltham, MA, USA) in nitrogen. The heating was from r.t. to 800 °C at a heating rate of 10 °C/min.

XRD analysis was conducted to assess the amorphous and crystalline phases of the mats; the scanning was at 2*θ* range from 5° to 60° in 0.02° steps with 1 s per step. The measurements were conducted at room temperature using a D8 Bruker Advance powder diffractometer (Billerica, MA, USA) with a LynxEye detector and filtered Cu Kα radiation (λ = 1.5406 Å).

The tensile tests of (Ch/HA)-*coat*-PLA, (Ch/HA)-*coat*-PLA/RA, [(Ch/RA)/HA]-*coat*-PLA, [(Ch/RA)/HA]-*coat*-PLA/RA, (HA/Ch)-*coat*-PLA, (HA/Ch)-*coat*-PLA/RA, [HA/(Ch/RA)]-*coat*-PLA and [HA/(Ch/RA)]-*coat*-PLA/RA fibrous materials were performed using INSTRON 3344 equipment (load cell of 50 N, stretching rate of 20 mm/min, at r.t.). Fibrous specimens (20 mm × 60 mm, width × length) were cut in so that their length aligned with the collector rotation direction. Thickness measurements were taken using a Digital Thickness Gauge FD 50 (Käfer GmbH, Bremen, Germany). Each specimen was clamped with an initial gauge length of 40 mm. Ten specimens per material were tested, and mean values for Young’s modulus, tensile strength, and elongation at break were determined.

The weight loss of Ch/HA- or HA/Ch-coated PLA or PLA/RA mats in PBS (pH 7.4) was estimated by immersion of the samples in the buffer solution for 24 h. After treatment, the mats were rinsed with distilled water and freeze-dried. The weight loss was then measured gravimetrically.

### 2.5. In Vitro RA Release Studies

The in vitro release of RA from the obtained RA-containing mats was carried out in PBS (pH 7.4, ionic strength of 0.1) at 37 °C for 1440 min. Samples consisting of 25 mg of PLA/RA mat ((Ch/HA)-*coat*-PLA/RA, [(Ch/RA)/HA]-*coat*-PLA/RA, (HA/Ch)-*coat*-PLA/RA or [HA/(Ch/RA)]-*coat*-PLA/RA mat) and 70 mg of [(Ch/RA)/HA]-*coat*-PLA or [HA/(Ch/RA)]-*coat*-PLA mat, were each placed in 100 mL of PBS and maintained in a shaker bath (JULABO SW23, Allentown, PA, USA) at 37 °C under continuous stirring at 100 rpm. The in vitro release studies were performed in such a way that the total amount of RA was one and the same in all samples. Periodically, 2 mL aliquots of the solution were taken for absorbance analysis at a wavelength of 324 nm (UV-Vis spectrophotometer, model DU 800, Beckman Coulter, Brea, CA, USA). An equivalent volume (2 mL) of fresh buffer solution was subsequently returned to the vial. Calculation of the released RA was performed using calibration curves exhibiting a correlation coefficient (R) of 0.999. All data are presented as the mean of three independent measurements. To determine the total content of RA in the tested fibrous materials, three fibrous samples of known weight were placed in 20 mL of ethanol and stirred for 6 h. The RA content was then measured spectrophotometrically using a DU 800 UV spectrophotometer (Beckman Coulter, Brea, CA, USA) at a wavelength of 327 nm.

### 2.6. Evaluation of the DPPH^•^ Radical Scavenging Activity of the Fibrous Materials

The antioxidant capacity of the RA and obtained fibrous materials was estimated using the DPPH^•^ radical scavenging assay. The procedure consisted of mixing 0.5 mL of RA solution in ethanol (concentration of 5 × 10^−3^ M) with 3 mL of ethanol DPPH^•^ solution (0.1 mM) in a test tube. 0.0099 g of PLA/RA mat, 0.0100 g of (HA/Ch)-*coat*-PLA/RA or (Ch/HA)-*coat*-PLA/RA mat, 0.0085 g of [HA/(Ch/RA)]-*coat*-PLA/RA mat, 0.0087 g of [(Ch/RA)/HA]-*coat*-PLA/RA mat, 0.0372 g of [HA/(Ch/RA)]-*coat*-PLA mat, or 0.0315 g of [(Ch/RA)/HA]-*coat*-PLA mat, with one and the same RA content of 0.0009 g, were placed in 3 mL of ethanol DPPH^•^ solution. After mixing, the samples were maintained in the dark for 30 min. The UV absorbance of the solutions was recorded at 517 nm using a DU 800 UV-Vis spectrophotometer (Beckman Coulter, Brea, CA, USA). The DPPH^•^ scavenging rate by the tested fibrous materials was calculated as follows:

Inhibition, AA, % = [(A_DPPH•_ − A_sample_)/A_DPPH•_] × 100 (1)

where A_DPPH•_ represents the absorbance of DPPH radical solution, and A_sample_ is the absorbance of the DPPH radical solution with the addition of the tested sample. All measurements were conducted in triplicate.

### 2.7. Estimation of the Antibacterial and Antifungal Properties of the Fibrous Materials

The antibacterial and antifungal activity of the (non)PEC-coated PLA fibrous materials containing RA against the bacteria *S. aureus* 3359 and *E. coli* 3397 and the fungi *C. albicans* 74 was studied using the viable cell-counting method. Tryptic Soy Agar medium (TSA, Becton Dickinson, Heidelberg, Germany) was used for the pre-culture of bacteria, and the incubation time was 24 h at 37 °C. Sabouraud Dextrose Agar medium (SDA, Becton Dickinson, Sparks, MD, USA) was used for the pre-culture of fungi *C. albicans* with incubation at 37 °C for 48 h. All studied mats were sterilized prior to the experiment using UV irradiation. Subsequently, they were exposed to a bacterial (fungal) suspension with a concentration of 10^5^ cells/mL prepared in test tubes containing 1 mL of saline solution (0.9%). The tubes were incubated at 37 °C for a duration of 24 h. Aliquots of 50 μL were collected from each test tube at specified time intervals (1, 3, and 24 h). Following tenfold serial dilution with sterile 0.9% saline solution, the samples were spread onto Petri dishes containing TSA (Becton Dickinson, Heidelberg, Germany) solid medium for tested bacteria and SDA (Becton Dickinson, Sparks, MD, USA) solid medium for tested fungi. The plates were incubated at 37 °C for 24 h (for bacteria) and for 48 h (for fungi). The colony-forming units (CFUs) of viable bacteria or fungi grown on the surface of the agar were counted. Each experiment underwent three separate counting trials.

### 2.8. Evaluation of S. aureus Cell Adhesion on the Surface of Fibrous Materials Using SEM

The interaction between pathogenic bacteria *S. aureus* 3359 and PLA mat and (non)coated with [HA/(Ch/RA)] or [(Ch/RA)/HA] PLA/RA mats was evaluated using SEM by observing bacterial cells adhered to the mat surfaces after exposure to a bacterial suspension. The mats were placed in 4.0 mL of *S. aureus* suspension (6.5 × 10^5^ cells/mL) and incubated at 37 °C for 24 h, followed by two washes with saline solution (0.9%) to eliminate the non-adhered cells. In order to fix the adhered bacteria, the mats were immersed in 4 mL of 2.5 vol% glutaraldehyde in saline solution at 4 °C for 5 h. Subsequently, the samples were rinsed with saline solution and freeze-dried. After gold coating, the morphology of *S. aureus* bacteria attached to the mats was analyzed by SEM (JEOL JSM-5510, Jeol Ltd., Tokyo, Japan).

### 2.9. Statistical Data Analysis

The results are presented as mean ± standard deviation (SD). One-way ANOVA was applied via GraphPad PRISM (Version 5, GraphPad Software Inc., San Diego, CA, USA) to determine statistical significance, with *p*-values of <0.05 (*), <0.01 (**), and <0.001 (***) indicating significant differences.

## 3. Results and Discussion

### 3.1. Preparation and Characterization of Polyelectrolyte Complex (PEC)-Coated PLA or PLA/RA Fibers

Schematic representation of the cross-sections of fibers from the newly developed RA-loaded fibrous materials with diverse design is shown in Scheme 1. The materials are divided into two series: PLA-based fibers (PLA series, left column of Scheme 1) and PLA/RA-based fibers (PLA/RA series, right column of Scheme 1). The coatings consist of PECs of Ch and HA and differ in the polyelectrolyte layer deposition sequence, as well as in the location of the loaded RA. The abbreviated designations of the both types of mats are also presented in Scheme 1.

PLA and PLA/RA fibrous materials were also prepared (Supplementary Materials, Scheme S1). These materials were obtained by electrospinning of a solution of the polymer alone or in the presence of the bioactive substance using a mixed solvent system DCM/DMF = 75/25 (*v*/*v*). In order to assess the effect of RA on the morphology of PLA/RA fibers, the content of the polyphenolic acid was selected to be 5 or 10 wt.% with respect to the polymer weight, and the mats are denoted by PLA/RA(5%) or PLA/RA(10%), respectively.

**Figure 1:**
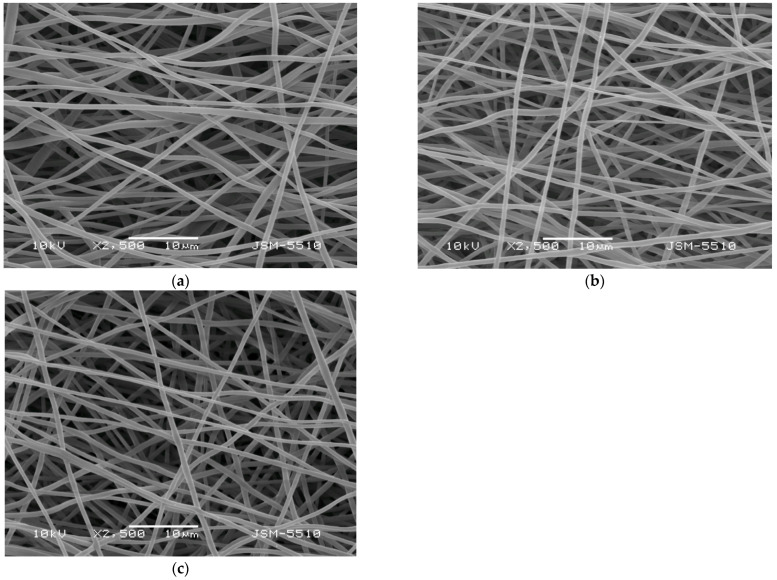
SEM micrographs of electrospun mats from: (**a**) PLA, (**b**) PLA/RA(5%) and (**c**) PLA/RA(10%).

SEM micrographs of PLA, PLA/RA(5%) and PLA/RA(10%) fibers are shown in Figure 1, and their mean diameters are listed in Table S1, Supplementary Materials. As seen from Figure 1, the fibers are defect-free. The PLA fibers have a mean diameter of 730 ± 220 nm. The loading of RA leads to an insignificant decrease in the mean fiber diameter value, and the latter is 660 ± 115 or 620 ± 130 nm for PLA/RA(5%) or PLA/RA(10%) mats, respectively. The decrease in the mean diameter of PLA/RA fibers with the increase in RA content might be attributed to the registered decrease in the viscosity of PLA spinning solutions in the presence of the low-molecular-weight compound RA (Supplementary Materials, Table S1). The detected viscosity reduction upon RA addition might be attributed to breaking up interchain interactions and increasing free volume. On the other hand, the conductivity of the spinning solution is not altered by the addition of RA (5 or 10 wt.%) (Supplementary Materials, Table S1). PLA and PLA/RA(10%) mats are used for the experiments by dip coating in order to prepare a coating from PEC between Ch and HA, and in a part of the experiments, RA is added to the Ch coating solution. Thus, RA content in the coated composite fibrous materials is not indicated in these sample abbreviations.

**Figure 2:**
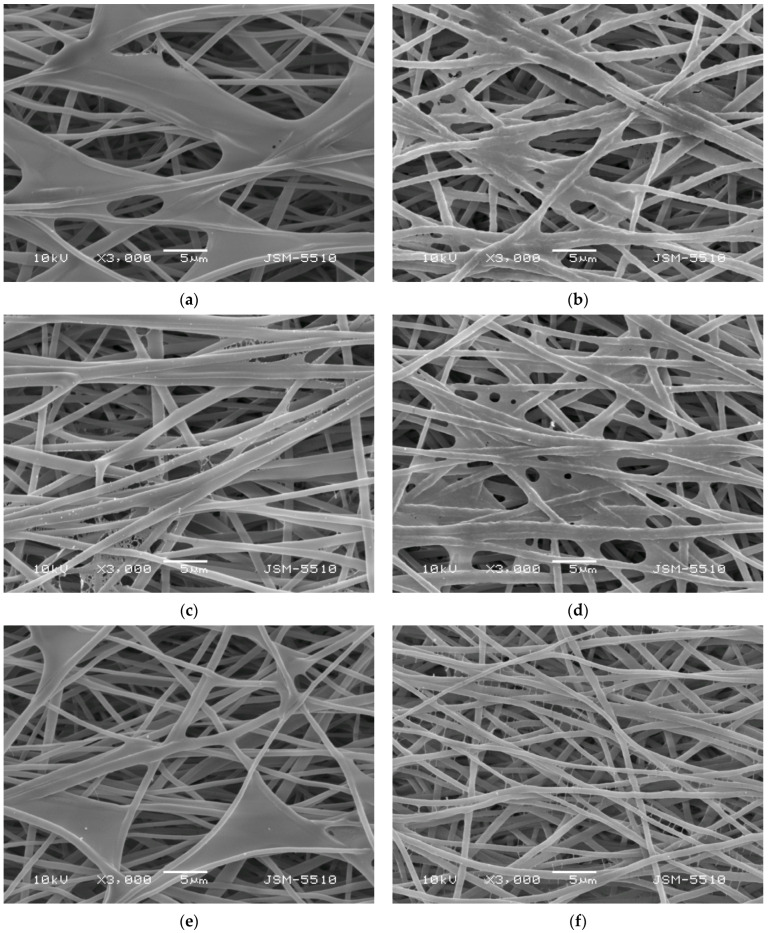

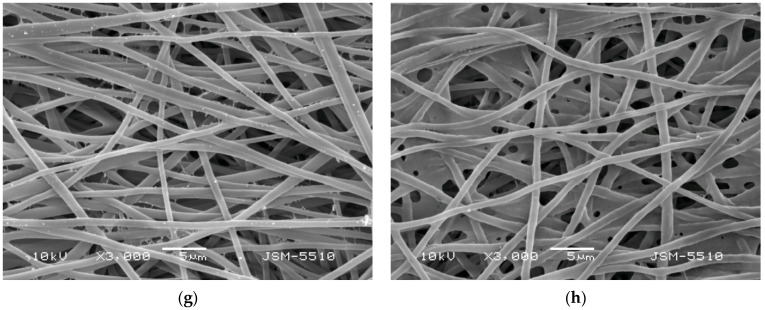
SEM micrographs of the coated electrospun mats: (**a**) (Ch/HA)-*coat*-PLA, (**b**) (Ch/HA)-*coat*-PLA/RA, (**c**) [(Ch/RA)/HA]-*coat*-PLA, (**d**) [(Ch/RA)/HA]-*coat*-PLA/RA, (**e**) (HA/Ch)-*coat*-PLA, (**f**) (HA/Ch)-*coat*-PLA/RA, (**g**) [HA/(Ch/RA)]-*coat*-PLA, and (**h**) [HA/(Ch/RA)]-*coat*-PLA/RA.

SEM micrographs of PLA or PLA/RA fibrous materials coated with PECs from Ch or Ch/RA and HA are shown in Figure 2. The deposition sequence of the oppositely charged polyelectrolytes during the dip coating procedure was varied using aqueous solutions of Ch or Ch/RA and HA. As seen from Figure 2, for all of the coated samples a thin film between the fibers is observed, which is due to the formation of a polymer coating.

The individual fibers also have a polymer coating on their surface, as evidenced by the increase in the mean fiber diameter value after the dip coating procedure (Supplementary Materials, Table S2).

**Figure 3:**
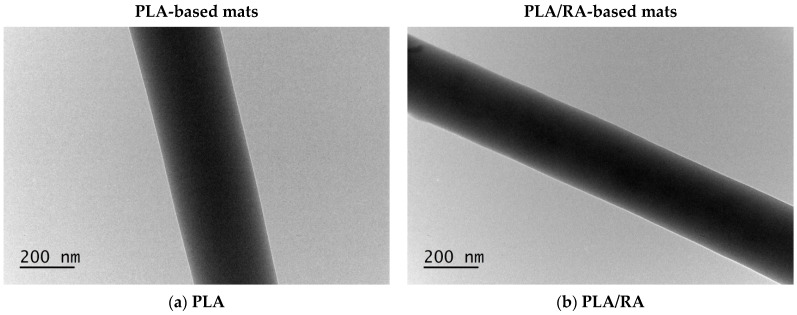

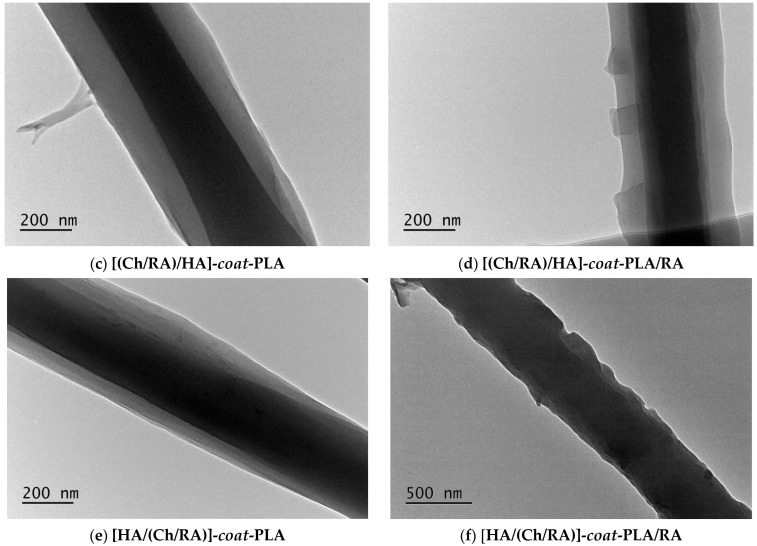
TEM micrographs of (non)coated electrospun fibers; magnification of ×20,000 (**a**–**e**) and ×10,000 (**f**).

The presence of a polymer coating on the surface of PLA or PLA/RA fibers was confirmed by TEM. As seen from Figure 3, PLA or PLA/RA fibers are monolithic and have a smooth surface. The formation of a coating from [(Ch/RA)/HA] or [HA/(Ch/RA)] led to obtaining fibers that exhibited an irregular surface morphology. Owing to the differences in density between PLA or PLA/RA and [(Ch/RA)/HA] or [HA/(Ch/RA)], the PEC coating appeared as a lighter outer layer surrounding the darker core of the PLA or PLA/RA fiber. The obtained SEM and TEM results indicated that RA-loaded fibrous materials with diverse design can be successfully fabricated by electrospinning, dip coating and subsequent PEC formation.

The stability of the coating from the PEC between Ch and HA on PLA mats in PBS with pH 7.4 was assessed by incubating the fibrous materials in this medium for 24 h. Since no alteration in the weight of the samples was detected, the results confirmed that Ch/HA or HA/Ch coating maintained its integrity under the tested conditions.

**Figure 4:**
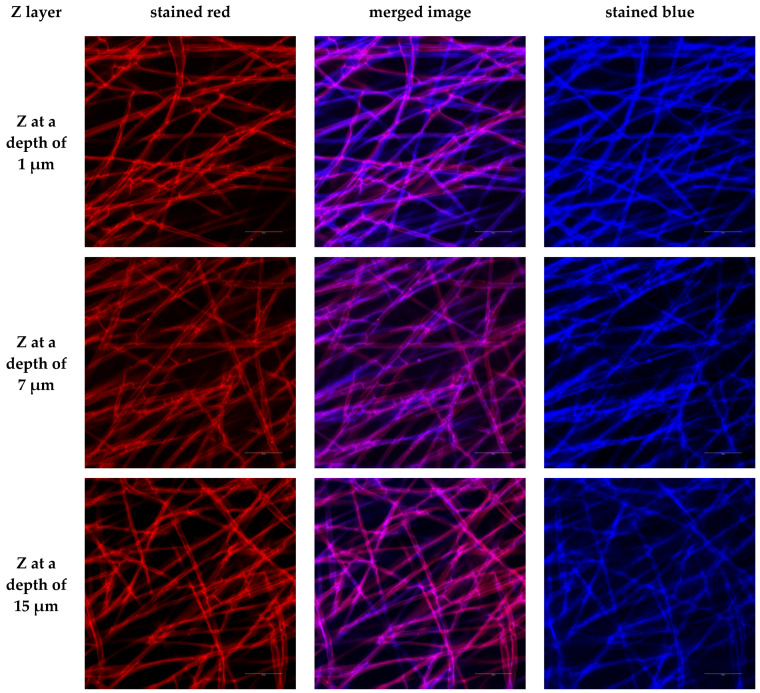
Confocal laser scanning microscopy (CLSM) images of fibers from [(Ch/RA)/(HA/RhB)]-*coat*-PLA. [(Ch/RA)/(HA/RhB)]-*coat*-PLA fibers are stained in red by the embedded RhB, and in blue by the embedded RA. Representative images of Z-layers at a depth of 1 µm, 7 µm, and 15 µm are shown. The Z step of the images is 1 μm. Scale bars = 5 µm.

Previously, the possibility for the application of CLSM as a non-destructive method for qualitative evidence of the presence of RA on the surface and in the bulk of electrospun materials has been demonstrated [31]. This is enabled by the fluorescent properties of RA (Supplementary Materials, Figure S1). In the present study, this analytical tool was applied in order to demonstrate the presence of the two polyelectrolyte partners on the surface of PLA or PLA/RA fibrous materials. RA was used as a fluorescent marker for the visualization of PLA/RA fibers (Supplementary Materials, Figure S1), as well as Ch/RA coating. For HA coating, it was visualized by the use of the fluorescent marker RhB. CLSM images of [(Ch/RA)/(HA/RhB)]-*coat*-PLA fibers are shown in Figure 4. [(Ch/RA)/(HA/RhB)] coating on the fibers consists of (Ch/RA) and (HA/RhB), as demonstrated by CLSM based on the observed blue and red, respectively, staining of the fibers in the CLSM images within a given Z-layer (Figure 4). This coating was observed not only on the surface but also in the bulk of the fibrous materials at a depth of 15 μm. For mats from [(Ch/RA)/(HA/RhB)]-*coat*-PLA/RA, CLSM analyses also demonstrated the presence of (Ch/RA) and (HA/RhB) in the formed coating on PLA/RA fibers (Supplementary Materials, Figure S2). Similar results were also obtained by CLSM analyses of [(HA/RhB)/(Ch/RA)]-*coat*-PLA (Supplementary Materials, Figure S3) and [(HA/RhB)/(Ch/RA)]-*coat*-PLA/RA (Supplementary Materials, Figure S4) fibrous materials. Overall, the results from the CLSM visualization showed that the dip coating technique enables the formation of a coating based on PEC between Ch and HA on PLA or PLA/RA fibers located on the surface and in the bulk of the fibrous material.

**Figure 5:**
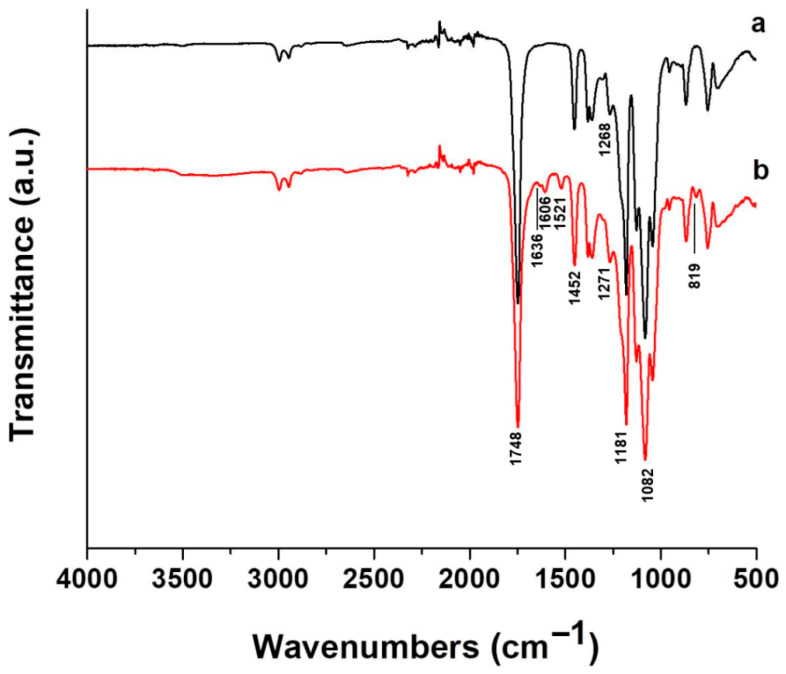

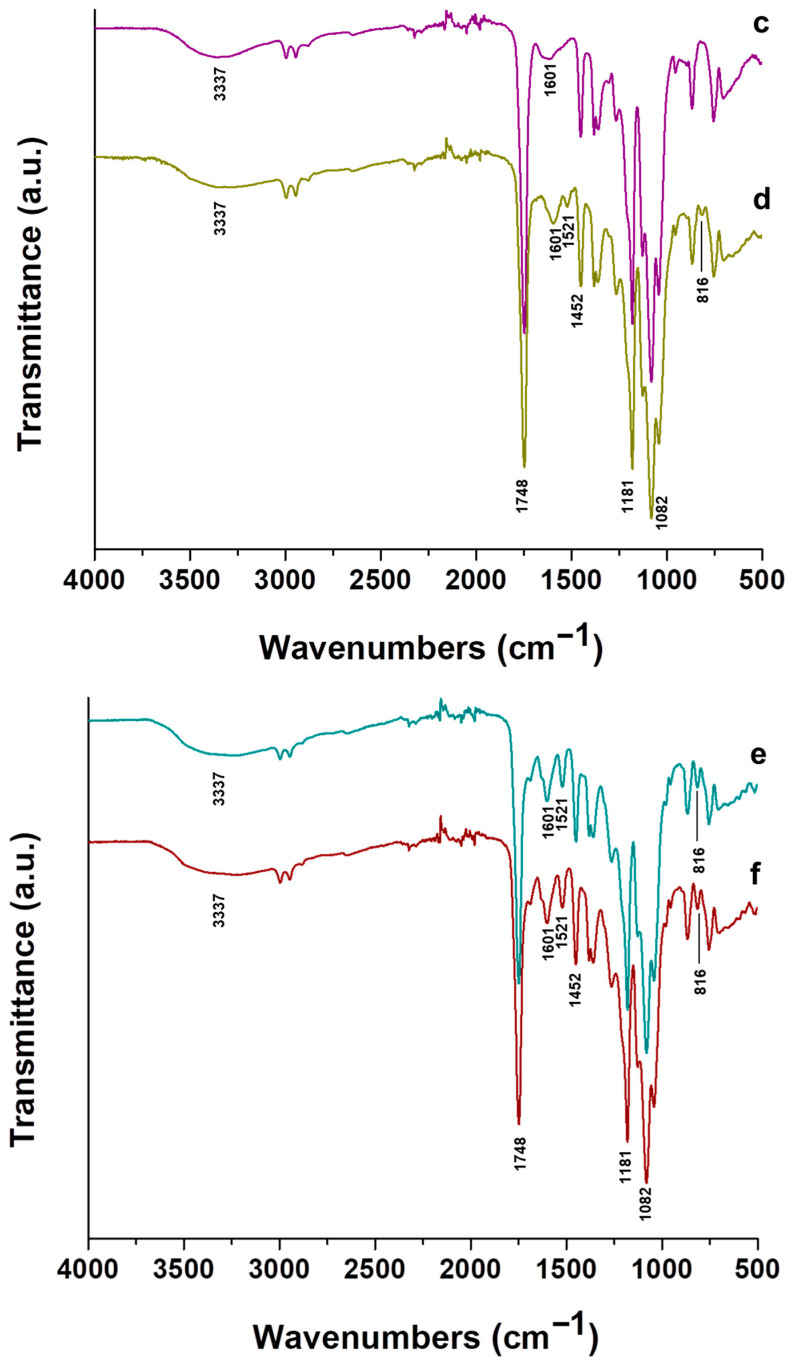
ATR–FTIR spectra of mats: (**a**) PLA, (**b**) PLA/RA, (**c**) (HA/Ch)-*coat*-PLA, (**d**) [HA/(Ch/RA)]-*coat*-PLA, (**e**) (HA/Ch)-*coat*-PLA/RA and (**f**) [HA/(Ch/RA)]-*coat*-PLA/RA.

ATR-FTIR spectroscopy was used to confirm the presence of the components constituting the studied (non)coated with PEC PLA and PLA/RA fibrous materials (Figure 5 and Supplementary Materials, Figure S6). The ATR-FTIR spectrum of PLA/RA mat (Figure 5b) displayed the characteristic absorption bands of PLA [33] (Figure 5a), namely: 1748 cm^−1^ corresponding to C=O stretching vibrations; 1452 cm^−1^ and 1382 cm^−1^ assigned to C–H bending vibrations of –CH(CH_3_) groups; and 1082 cm^−1^ attributed to C–O–C stretching vibrations. In addition, bands characteristic of RA were observed at 1636 cm^−1^ (C=C and C=O stretching vibrations), 1606 cm^−1^ (C=O and C=C stretching vibrations), 1521 cm^−1^ (C–C stretching in the aromatic ring), and 819 cm^−1^ [out-of-plane bending vibrations of CH and OH groups (both in ring and aliphatic ones)] [34]. In the PLA/RA mat spectrum (Figure 5b), several characteristic bands were shifted compared to those of RA (Supplementary Materials, Figure S5c): the band at 1636 cm^−1^ shifted by 11 cm^−1^ toward lower wavenumbers, the band at 1606 cm^−1^ shifted by 1 cm^−1^ toward lower wavenumbers, and the band at 1521 cm^−1^ shifted by 6 cm^−1^ toward higher wavenumbers. These shifts suggested the presence of specific intermolecular interactions, most likely hydrogen bonding, between PLA and RA. The results are consistent with those reported by other authors for polyester fibrous materials containing polyphenolic acids (caffeic acid, ferulic acid) [35,36]. In the ATR-FTIR spectra of PEC Ch/HA- or HA/Ch-coated PLA mats (Figure 5c and Supplementary Materials, Figure S6a), in addition to the characteristic PLA bands, a broad band at 3337 cm^−1^ was observed, attributed to O–H and N–H stretching vibrations from the PEC coating [37]. Another broad band with a maximum at 1601 cm^−1^ assigned to asymmetric COO^−^ stretching vibrations of HA in the PEC coating was recorded [37]. Furthermore, in the spectra of [HA/(Ch/RA)]-*coat*-PLA and [(Ch/RA)/HA]-*coat*-PLA mats (Figure 5d and Supplementary Materials, Figure S6b), additional bands at 1521, and 816 cm^−1^ were detected, confirming the presence of RA [34] incorporated into the PEC coating. As seen from Figure 5e,f and Figure S6c,d (Supplementary Materials), in the spectra of PLA/RA mats coated with PEC from (HA/Ch) or (Ch/HA) or PEC from [HA/(Ch/RA)] or [(Ch/RA)/HA], the bands ascribed to the RA overlapping with the bands characteristic for PLA and PEC coating.

Therefore, the results obtained from ATR-FTIR analyses proved the successful incorporation of RA in the bulk of fibers and/or in the PEC coating onto the fibers.

**Figure 6:**
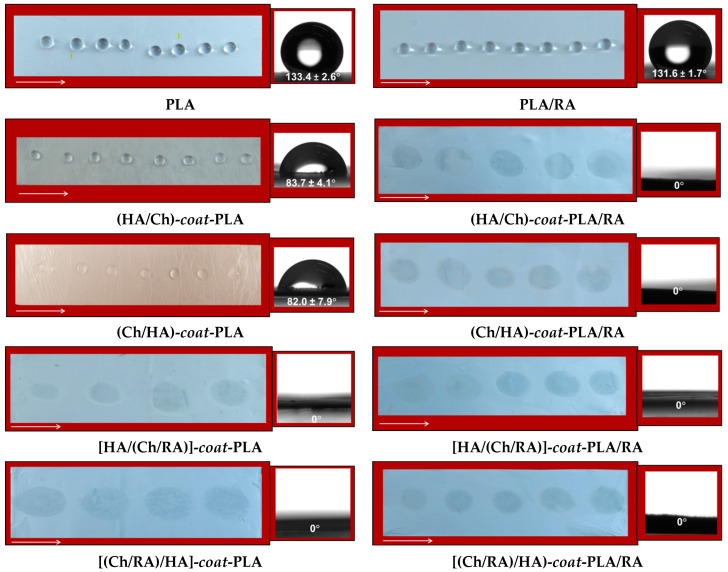
Digital photographs of water droplets placed on the surface of fibrous mats; the water contact angle value is indicated in the images. The collector rotation direction is presented by white arrow.

The wetting characteristics of PEC-coated PLA or PLA/RA mats were investigated. As seen from Figure 6, the PLA mat is hydrophobic, with a water contact angle of ca. 133°. It was found that the incorporation of RA in the PLA mat by electrospinning of a blend solution of the polymer and the bioactive compound did not alter the hydrophobic character of the non-woven textile. The formation of PEC from HA/Ch or Ch/HA onto the PLA mat—(HA/Ch)-coat-PLA or (Ch/HA)-coat-PLA mats—led to a certain increase in the surface hydrophilicity of the fibrous material with water contact angles of ca. 83°. For PEC-coated (HA/Ch)-*coat*-PLA/RA and (Ch/HA)-*coat*-PLA/RA mats, a water contact angle of 0° was registered. The difference in water contact angle values between the PEC-coated PLA and PLA/RA mats might be attributed to the presence of RA in the structure of the coated PLA/RA electrospun fibers.

In the case of PEC-coated PLA-based mats, there are no ionic interactions between the aliphatic polyester and the functional groups of Ch or HA during the formation of the first polyelectrolyte layer. The deposition of the second layer, composed of the oppositely charged polyelectrolyte, results in the formation of a PEC in which the majority of the ionized carboxyl groups of HA or amino groups of Ch are engaged in the complex. This might explain the partial surface hydrophilization of (HA/Ch)-*coat*-PLA and (Ch/HA)-*coat*-PLA fibrous materials (Figure 6). For (HA/Ch)-*coat*-PLA/RA and (Ch/HA)-*coat*-PLA/RA mats, RA molecules on the surface of the electrospun fibers are able to interact with Ch or HA by the formation of ionic or hydrogen bonds during the deposition of the first polyelectrolyte layer. This interaction might lead to the formation of “defects” along the polyelectrolyte chains that do not participate in PEC formation during the deposition of the second layer of the oppositely charged partner. This, in turn, allows the deposited second layer to contain free ionized groups that are unreacted with the first layer, which can lead to surface hydrophilization of the fibrous material, reaching water contact angle values of 0°, as registered for the (HA/Ch)-*coat*-PLA/RA and (Ch/HA)-*coat*-PLA/RA mats. The hydrophilic–hydrophobic behavior of PLA or PLA/RA mats coated with the RA-containing PEC might be interpreted in a similar manner. RA is incorporated into the Ch-based coating. In the process of deposition of the Ch/RA layer, some of the protonated amino groups of chitosan might be engaged in ionic interaction with RA and not be able to participate in PEC formation with HA. This leads to the presence of free ionized carboxyl groups from HA that impart hydrophilicity to the fibrous materials, as evidenced for [HA/(Ch/RA)]-*coat*-PLA, [(Ch/RA)/HA]-*coat*-PLA, [HA/(Ch/RA)]-*coat*-PLA/RA, and [(Ch/RA)/HA]-*coat*-PLA/RA mats having water contact angle values of 0° (Figure 6). The obtained findings are consistent with previous reports demonstrating the enhanced hydrophilicity of materials in the presence of polar groups on the material surface [38,39,40,41]. The presence of highly hydrophilic OH groups from the HA on the fiber surface may also contribute to the observed hydrophilicity of the mats [39,40]. Multiple hydroxyl groups attached to an aromatic ring are present in the RA structure, and they may also enhance the hydrophilicity of the fibrous materials. In addition, the registered decrease in the water contact angle of PEC-coated fibrous materials containing RA might also be attributed to the surface roughness of the prepared fibrous materials. According to previous reports, surface roughness affects the hydrophilic behavior of materials [39,42].

Regarding potential drug delivery applications of the developed materials, the hydrophilicity of the mats is an essential characteristic for ensuring a fast therapeutic response of the drug. Moreover, measuring the water contact angle is important for clarifying how the composition of the mats relates to their potential use as wound dressings. It is anticipated that fibrous materials coated with RA-containing PEC exhibiting contact angles of 0° will be particularly suitable as dressing materials for treating open wounds with higher levels of exudate leakage. Additionally, the mats loaded with RA will be appropriate for use in dressings intended for infected wounds.

**Figure 7:**
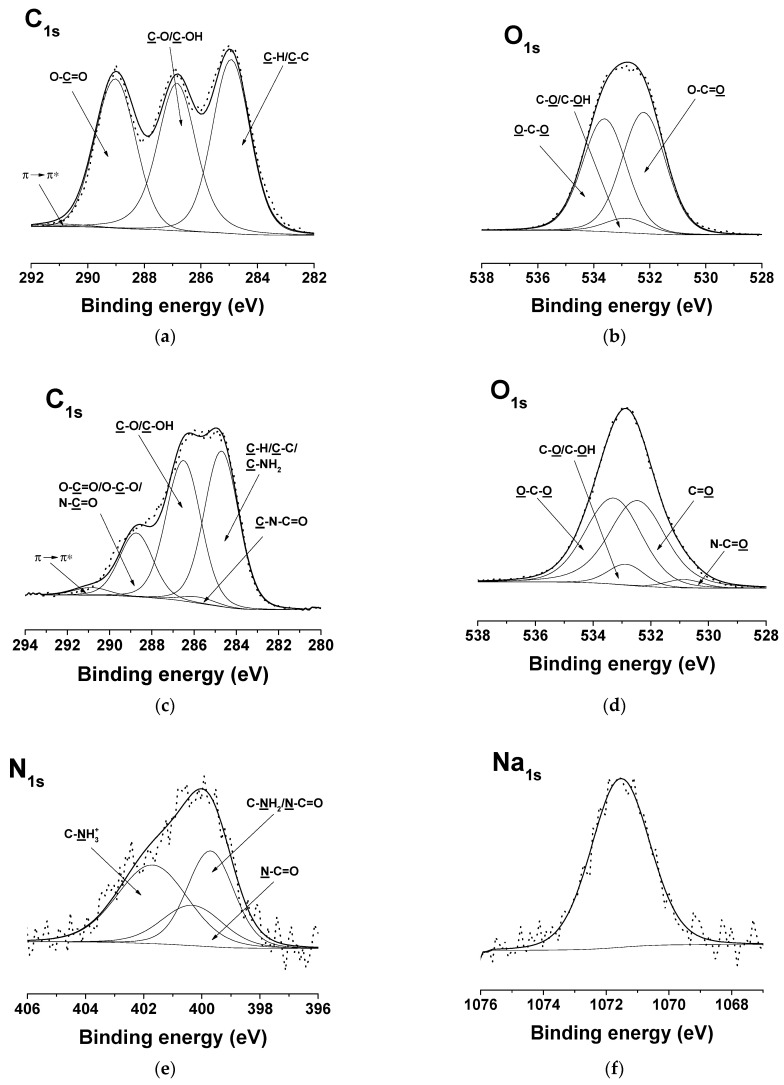

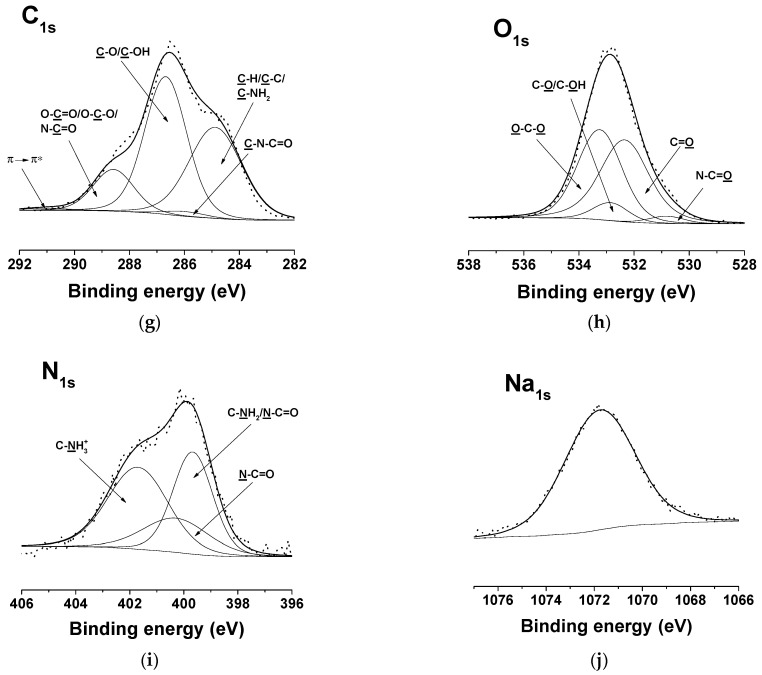
XPS peak fittings for PLA/RA mat [(**a**) C_1s_, (**b**) O_1s_], (HA/Ch)-*coat*-PLA/RA mat [(**c**) C_1s_, (**d**) O_1s_, (**e**) N_1s_, (**f**) Na_1s_] and [HA/(Ch/RA)]-*coat*-PLA/RA mat [(**g**) C_1s_, (**h**) O_1s_, (**i**) N_1s_, (**j**) Na_1s_].

The surface composition of PLA mats coated with PEC Ch/HA (HA/Ch) or (Ch/RA)/HA [HA/(Ch/RA)], as well as of RA-containing PLA mats, was monitored by XPS. The C_1s_ signal of the PLA mat displayed three components, i.e., a peak at 285.0 eV ascribed to C-H/C-C, a peak assigned to C-O at 286.7 eV and a peak at 288.9 eV for O-C=O (Supplementary Materials, Figure S7a) [43]. In the detailed O_1s_ spectrum two peaks were detected—a peak at 533.5 eV assigned to C-O and a peak at 532.1 eV characteristic of O-C=O (Supplementary Materials, Figure S7b) [43]. Four peaks were detected in the C_1s_ spectra of PEC Ch/HA (HA/Ch)-coated PLA fibrous materials (Supplementary Materials, Figures S7c and S8a). The peak at 285.0 eV was assigned to C-H/C-C of PLA, Ch and HA, and to C-NH_2_ of Ch; the peak at 286.2 eV was characteristic for C-N-C=O of Ch and HA; the peak at 286.6 eV was attributed to C-O of PLA, C-O/C-OH of Ch and HA; the peak at 288.6 eV was ascribed to O-C=O of PLA, O-C-O and N-C=O of Ch and O-C=O, O-C-O and N-C=O of HA. Compared to the O_1s_ spectrum of PLA mat (Supplementary Materials, Figure S7b), in addition to the peaks at 533.3 eV corresponding to C-O of PLA, O-C-O of Ch and HA, and at 531.9 eV characteristic for O-C=O of PLA and C=O and COO^−^ of HA, two new peaks at 532.5 eV for C-OH of Ch and HA and at 530.5 eV for N-C=O of Ch and HA were recorded in the O_1s_ spectra of PEC-coated mats (Supplementary Materials, Figures S7d and S8b). In the spectra of the same fibrous materials, the appearance of the two peaks in the N_1s_ region (consisting of three components—at 399.3 eV for N-C=O and C-NH_2_ groups from Ch [44], at 400.0 eV for N-C=O from HA [45] and at 401.1 eV for NH_3_^+^ from Ch [44]) (Supplementary Materials, Figures S7e and S8c), as well as in the Na_1s_ region at 1071.2 eV (Supplementary Materials, Figures S7f and S8d), was detected. The presence of N_1s_ peaks and Na_1s_ peaks additionally proved the simultaneous presence of both polyelectrolytes on the PEC-coated surface of the fibers. Compared to the detailed C_1s_ spectra of Ch/HA (HA/Ch)-coated PLA mats (Supplementary Materials, Figures S7c and S8a), in the C_1s_ spectra of PLA mats coated with (Ch/RA)/HA or HA/(Ch/RA) (Supplementary Materials, Figures S7g and S8e) the appearance of a new peak at 290.9 eV ascribed to the π → π* shake-up satellite from the aromatic ring of RA was recorded [31]. Four components were observed in the O_1s_ spectra—at 533.1 eV (C-O of PLA, O-C-O of Ch and HA), at 532.5 eV (C-OH of Ch and HA and C-O/C-OH of RA), at 531.8 eV (O-C=O of PLA and RA and C=O and COO^−^ of HA) and at 530.5 eV (N-C=O of Ch and HA) (Supplementary Materials, Figures S7h and S8f). In the spectra of the same mats, the N_1s_ peak and Na_1s_ peak (1071.3 eV) were detected (Supplementary Materials, Figures S7i,j and S8g,h). The N_1s_ peak showed the presence of three components, at 399.5 eV for N-C=O and C-NH_2_ groups from Ch, at 400.2 eV for N-C=O from HA and at 401.6 eV for NH_3_^+^ from Ch. The N_1s_ and Na_1s_ spectra of the (Ch/RA)/HA- or HA/(Ch/RA)-coated PLA mats also revealed the presence of Ch and HA on the fiber surface. In the C_1s_ signal of the PLA/RA mat compared to those of PLA mat, a new component at 290.9 eV assigned to the π → π* shake-up satellite from the RA ring was detected (Figure 7a). The other three components were detected at 285.0 eV (C-H/C-C of PLA and RA), at 286.9 eV (C-O of PLA, C-O/C-OH of RA) and at 288.9 eV (O-C=O of PLA and RA). A new peak at 532.5 eV ascribed to C-O/C-OH of RA was recorded in the detailed O_1s_ spectrum of the PLA/RA mat (Figure 7b). These peaks are in accordance with previously reported literature data and match well with the structures of the PLA [43] and RA [31] present in the mat. In the XPS spectra of PLA/RA mats coated with PEC of Ch/HA (HA/Ch) or (Ch/RA)/HA (HA/(Ch/RA)), new peaks for N_1s_ and Na_1s_ in addition to the peaks for C_1s_ и O_1s_ were registered (Figure 7c–j, Supplementary Materials, Figure S9). In the detailed N_1s_ spectrum, three components—the first ascribed to N-C=O and C-NH_2_ from Ch at 399.7 eV, the second assigned to N-C=O from HA at 400.3 eV and the third characteristic for NH_3_^+^ from Ch at 401.7 eV—were detected (Figure 7e,i, Supplementary Materials, Figure S9c,g). Differences in the detailed C_1s_ and O_1s_ spectra of the same fibrous materials (Figure 7c,d,g,h, Supplementary Materials, Figure S9a,b,e,f) were also identified compared to those of the PLA/RA mat (Figure 7a,b). In the C_1s_ spectra, a peak at 286.1 eV corresponding to C-N-C=O of Ch and HA appeared (Figure 7c,g, Supplementary Materials, Figure S9a,e). Four components were also detected in the C_1s_ spectra: the peak at 285.0 eV ascribed to C-H/C-C of PLA, RA, Ch and HA; the peak at 286.7 eV assigned to C-O of PLA, C-O/C-OH of RA, Ch and HA; the peak at 288.8 eV for O-C=O of PLA and RA, O-C-O and N-C=O of Ch and O-C=O, O-C-O and N-C=O of HA; and the peak at 291.0 eV associated with the π → π* shake-up satellite from the RA ring. In the expanded O_1s_ spectra, a new peak at 530.8 eV assigned to N-C=O of Ch and HA with the exception of the peaks at 533.3 eV (C-O of PLA, O-C-O of Ch and HA), at 532.9 eV (C-OH of Ch and HA, C-O/C-OH of RA) and at 532.3 eV (O-C=O of PLA and C=O and COO^-^ of HA, C=O/O-C=O of RA), was detected (Figure 7d,h, Supplementary Materials, Figure S9b,f).

Overall, the results from the XPS analyses performed revealed both the successful formation of a PEC coating between Ch and HA on the fiber surface, as well as the successful incorporation of RA into the fibers and/or into the PEC coating on the fibers.

The XRD analysis showed that PLA in the electrospun materials composed solely of this semi-crystalline polymer is in an amorphous state (Supplementary Materials, Figure S10). The lack of a crystalline phase is attributed to the rapid solidification of PLA during the electrospinning process, along with its low tendency to crystallize. Regarding RA, it was found that it was also present in an amorphous state both in the PLA/RA fibers and in the RA-containing coatings. The PEC coatings (Ch/HA or HA/Ch) are likewise amorphous, which is consistent with previous reports [22,46]. In conclusion, the results obtained from XRD analysis indicated that all components of the composite fibrous materials are in an amorphous state.

Figure S11 (Supplementary Materials) presents TGA thermograms of PLA or PLA/RA fibrous materials coated with PEC between Ch and HA. For comparison, the thermograms of RA powder, PLA mat and PLA/RA mat are also shown. RA powder degrades thermally at three stages in the temperature range from 240 to 380 °C, from 380 to 448 °C (maximum degradation temperature T_d_^max^ 392 °C) and from 448 °C to 800 °C; however, the degradation is incomplete, as evidenced by the detected residual mass of ca. 39% after heating to 800 °C. The obtained results are in good agreement with the literature data [47,48]. In accordance with previous reports [49,50], PLA degrades thermally at one stage in the range from 300 to 400°C (T_d_^max^ = 391 °C) without recording any residual mass after heating to 800 °C. The formation of PEC coating between Ch and HA on PLA fibrous material, namely, (Ch/HA)-*coat*-PLA and (HA/Ch)-*coat*-PLA mats, led to a certain decrease in the thermal stability of the fibrous materials compared to the PLA mat with T_d_^max^ at 376 and 378 °C, respectively (Supplementary Materials, Figure S11a). According to the literature, the Ch/HA complex exhibited a maximum degradation temperature of about 280 °C [51]. The reduced thermal stability observed in PEC-coated PLA mats compared with PLA mats was most likely attributed to the presence of a Ch/HA coating with lower thermal stability on the fiber surface. The presence of RA in the PEC coating of PLA fibers did not significantly alter the thermal stability of the mats compared to that of (Ch/HA)-*coat*-PLA and (HA/Ch)-*coat*-PLA fibrous materials (Supplementary Materials, Figure S11a), with T_d_^max^ at 380 and 374 °C for [(Ch/RA)/HA]-*coat*-PLA and [HA/(Ch/RA)]-*coat*-PLA mats, respectively. It was found that the incorporation of RA into PLA fibers by electrospinning of a blend solution of the both components resulted in PLA/RA mat that exhibited lower thermal stability than that of PLA mat (Supplementary Materials, Figure S11b), with T_d_^max^ at 374 °C for the PLA/RA mat. The detected lower thermal stability might be due to a decrease in the PLA crystallinity upon addition of RA [52]. The formation of PEC coating between Ch and HA in (Ch/HA)-*coat*-PLA/RA and (HA/Ch)-*coat*-PLA/RA mats did not lead to a significant alteration of the thermal stability, with T_d_^max^ at 376 and 372 °C, respectively. The presence of RA in the PEC coating on PLA fibers also did not significantly affect the thermal stability, with T_d_^max^ values of 384 and 375 °C for [(Ch/RA)/HA]-*coat*-PLA/RA and [HA/(Ch/RA)]-*coat*-PLA/RA mats, respectively. Therefore, the obtained TGA results elucidated that the formation of PEC coating between Ch and HA, as well as the incorporation of RA into PLA fibers, led to a certain decrease in the thermal stability of PLA fibers. The presence of RA in the PEC coating did not have a significant effect on the thermal stability of the respective fibrous materials.

**Figure 8:**
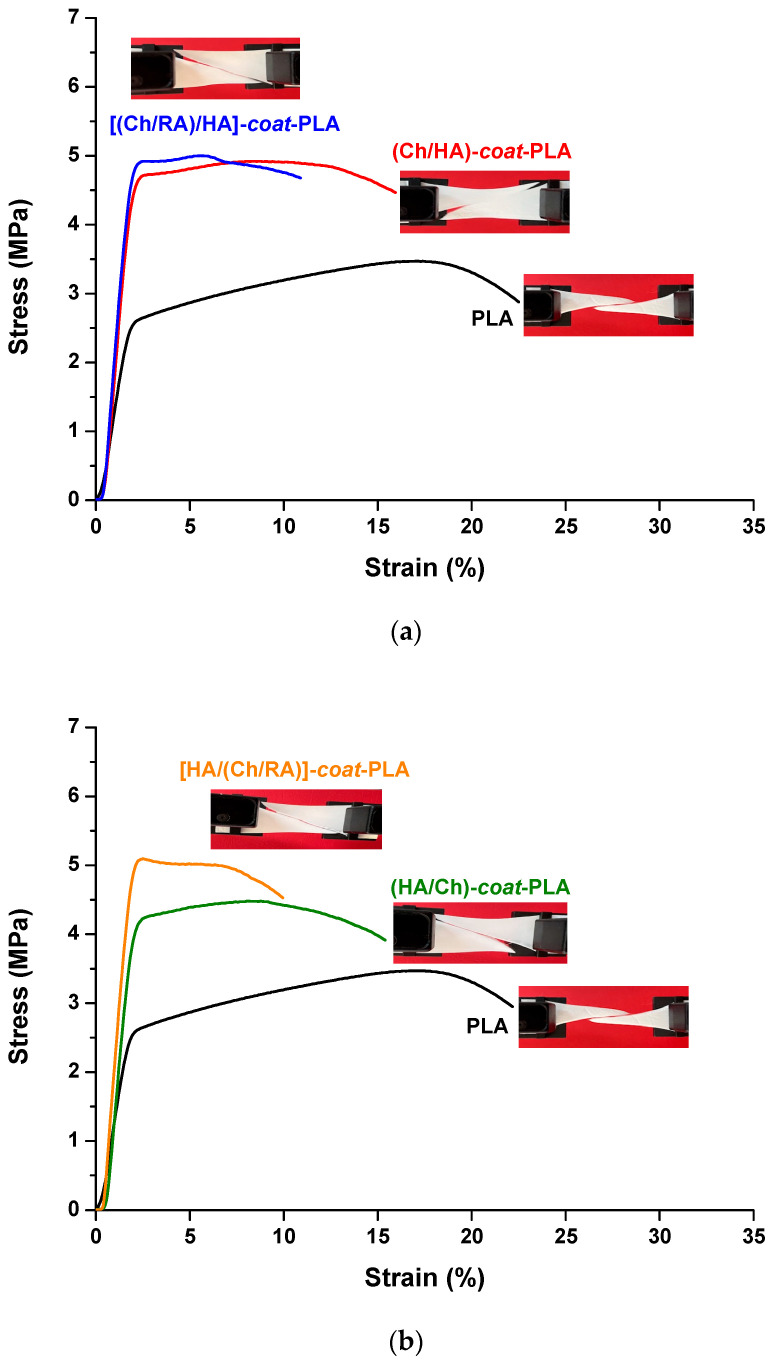
Stress–strain curves of PLA-based fibrous materials: (**a**) [(Ch/RA)/HA]-*coat*-PLA, (Ch/HA)-*coat*-PLA and PLA mats, and (**b**) [HA/(Ch/RA)]-*coat*-PLA., (HA/Ch)-*coat*-PLA and PLA mats.

The fibrous materials were subjected to tensile tests, and the recorded values of their tensile parameters are listed in Table S3 (Supplementary Materials). Figure 8 shows stress–strain curves of PLA-based fibrous materials. PLA mats were characterized by a tensile strength of about 3.5 MPa, Young’s modulus of 193 MPa, and an elongation at break of about 20%. The formation of the PEC coating from Ch and HA on a PLA mat, regardless of the deposition sequence of the polycation and polyanion, led to the preparation of fibrous materials with higher values of tensile strength (up to ~5.0 MPa) and Young’s modulus (in the range of 320–350 MPa), as well as lower elongation at break (in the range of 15–18%) compared to the PLA mat. The obtained results are consistent with previous reports on improving the tensile strength and Young’s modulus of electrospun materials from polyhydroxybutyrate when coated with a thin layer of chitosan or cellulose acetate [53]. The improved mechanical performance might be attributed to the formation of a thin film not only on the surface of the fibers but also between the fibers, as evidenced by SEM analysis. The presence of RA in the chitosan coating did not significantly affect the tensile characteristics of PLA fibrous materials (Figure 8 and Supplementary Materials, Table S3). PLA/RA fibrous materials had tensile characteristics similar to those of PLA mats (Supplementary Materials, Figure S12 and Table S3). Similarly to PLA mats coated with the complex, the formation of the PEC coating from Ch and HA on PLA/RA fibrous materials led to a certain increase in tensile strength to values in the range of 4.2–4.6 MPa, Young’s modulus in the range of 190–260 MPa and 15–20% elongation at break. These values were slightly lower than those determined for PLA fibrous materials coated with PECs from Ch and HA. The tensile test results for the coated PLA or PLA/RA mats indicated that these newly developed fibrous materials possessed mechanical characteristics that fell within the range observed for human skin (skin tensile strength from 1 to 32 MPa, a Young’s modulus from 2.9 to 150 MPa and an elongation at break from 17% to 207% [54]), suggesting their suitability for application as wound dressings. The obtained results also demonstrated the effectiveness of the dip coating technique for the preparation of PEC coatings from Ch or (Ch/RA) and HA on PLA or PLA/RA fibrous materials.

### 3.2. In Vitro Release Studies of RA from (Non)coated PLA or PLA/RA Mats

**Figure 9:**
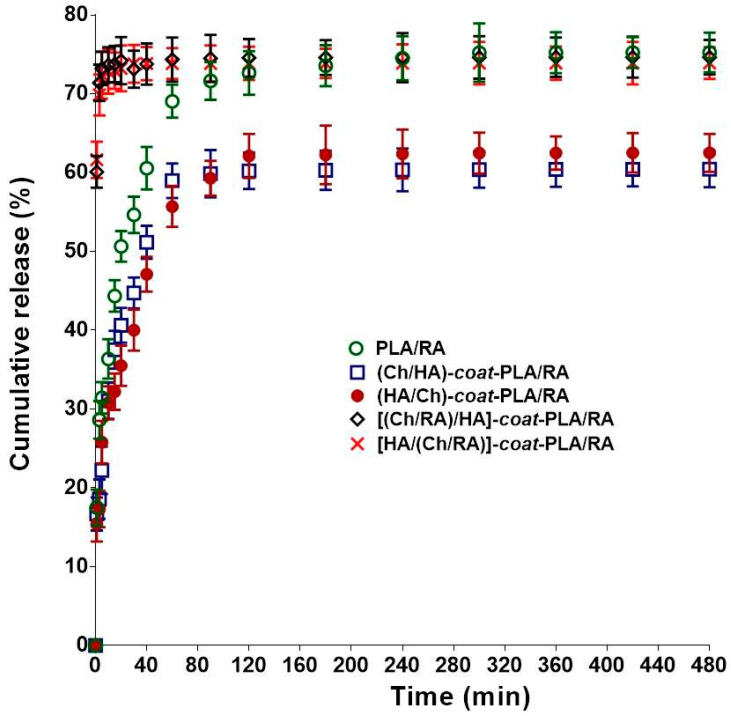
Release profiles of RA from mats: PLA/RA, (Ch/HA)-*coat*-PLA/RA, (HA/Ch)-*coat*-PLA/RA, [(Ch/RA)/HA]-*coat*-PLA/RA and [HA/(Ch/RA)]-*coat*-PLA/RA in PBS (pH 7.4, I = 0.1); 37 °C.

The release of RA from (non)coated PLA or PLA/RA mats was evaluated in vitro using PBS (pH 7.4, I = 0.1) at 37 °C, and the release time was 1440 min. Since it has been reported that PLA degrades slowly under the same conditions, remaining intact within more than 10,000 h [55], any interference of PLA degradation products should be excluded. As shown in Figure 9, the RA release profile from PLA/RA-based fibrous materials depends on the absence/presence of the PEC coating, as well as on the absence/presence of RA in the coating. It was found that ca. 61% of the incorporated RA in PLA/RA mats was released within the initial 40 min, increasing to 75.3% at 480 min. The amount of the released RA remained unchanged up to 1440 min. The presence of PEC (Ch/HA or HA/Ch) coating in (Ch/HA)-*coat*-PLA/RA and (HA/Ch)-*coat*-PLA/RA mats led to a slower RA release. About 51% and 47% of the released RA was registered within the first 40 min for (Ch/HA)-*coat*-PLA/RA and (HA/Ch)-*coat*-PLA/RA fibrous materials, respectively. In the case of PEC-coated PLA/RA mats, up to ca. 60% of RA was released over 480 min. The observed slower release of RA is most likely due to the presence of a film from the complex on the fiber surface, through which RA incorporated in the fibers has to diffuse before release. This is consistent with findings reported in other studies by some of the authors [22,56].

The fastest release of RA was observed in the cases of mats with RA incorporated in the PEC coating—[(Ch/RA)/HA]-*coat*-PLA/RA and [HA/(Ch/RA)]-*coat*-PLA/RA mats. Approximately 73% and 72% of RA were released within the first 5 min., reaching 75% and 74%, respectively, at 480 min. This rapid initial release is attributed to the fast diffusion of RA incorporated in the complex through the PEC coating layer deposited on the fiber surface. In the case of PLA-based mats with RA incorporated in the coating: [(Ch/RA)/HA]-*coat*-PLA and [HA/(Ch/RA)]-*coat*-PLA, the bioactive compound was found to release rapidly, with ca. 96% released within the first 5 min and about 99% after 480 min (Supplementary Materials, Figure S13). Furthermore, for all coated PLA mats or (non)coated PLA/RA mats, the RA incorporated either in the coating or in the fibers is in an amorphous state, and the mats exhibit hydrophilic behavior, which contributes to the increased release rate of RA in the buffer solution.

The fabricated RA-loaded fibrous materials exhibited a release profile, characterized by an initial rapid release of RA followed by a subsequent phase of gradual release. An initial rapid RA release is advantageous for suppressing bacterial growth in infected wounds, while a gradual release is essential to inhibit the further proliferation of residual bacterial cells. The fast initial release behavior of RA from PEC/RA-*coat*-PLA or PEC/RA-*coat*-PLA/RA mats renders these materials promising carriers of bioactive compounds, enabling the attainment of a rapid biological response. The observed slower release of RA from PEC-coated PLA/RA mats suggests that these materials are likewise suitable as systems for achieving sustained biological effect. Accordingly, the developed fibrous mats are appropriate for application in wound care and drug delivery.

### 3.3. Antioxidant Properties of the Fibrous Materials

Electrospun fibrous materials loaded with compounds with antioxidant properties intended for wound healing applications offer a promising strategy to regulate reactive oxygen species (ROS) levels and restore redox balance, while supporting tissue regeneration [57,58].

**Figure 10:**
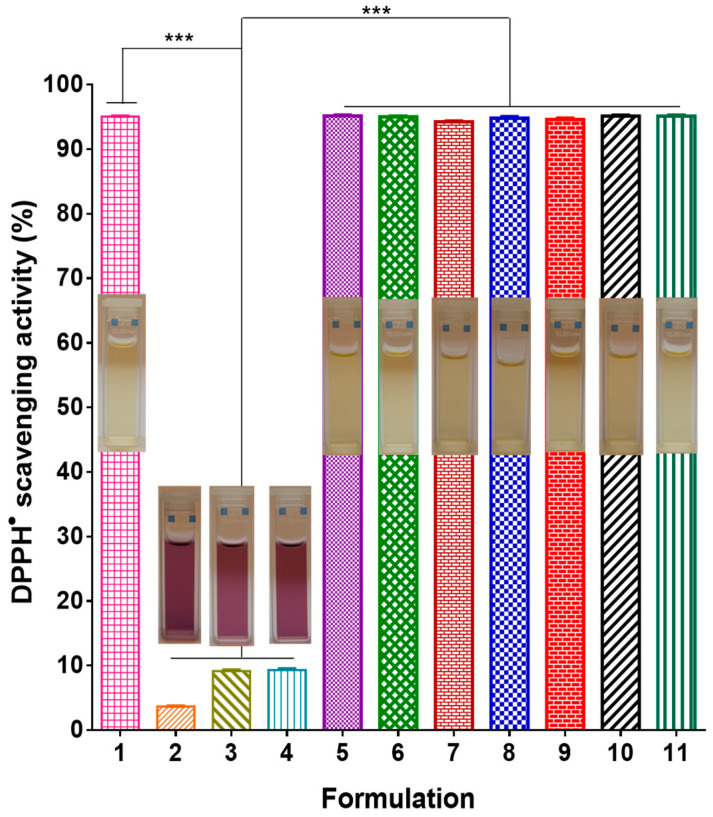
Antioxidant activity of: 1—RA solution, 2—PLA mat, 3—(HA/Ch)-*coat*-PLA, 4—(Ch/HA)-*coat*-PLA, 5—[HA/(Ch/RA)]-*coat*-PLA, 6—[(Ch/RA)/HA]-*coat*-PLA, 7—PLA/RA, 8—(HA/Ch)-*coat*-PLA/RA, 9—(Ch/HA)-*coat*-PLA/RA, 10—[HA/(Ch/RA)]-*coat*-PLA/RA, and 11—[(Ch/RA)/HA]-*coat*-PLA/RA. Digital images of the solutions are displayed in the bars. *** *p* < 0.001.

Therefore, the antioxidant activity of RA-loaded fibrous materials, as well as those coated with Ch/HA or HA/Ch complexes, or with (Ch/RA)/HA or HA/(Ch/RA) complexes, was investigated by UV-Vis spectrophotometry against the DPPH^•^ free radical. The decrease in the absorbance of the DPPH^•^ solution at λ = 517 nm was monitored over 30 min in the presence and in the absence of the fibrous materials. For comparison, the activity of non-coated and PEC-coated PLA mats was also evaluated. PLA fibrous materials displayed very low activity, reducing the absorbance of DPPH^•^ by only 3.7 ± 0.1% (Figure 10). 

The PEC-coated PLA mats also demonstrated a weak DPPH^•^ scavenging ability—the absorbance of DPPH^•^ dropped by ca. 9.2%. High antioxidant activity was detected in the case of RA-containing fibrous materials. After 30 min of contact with these mats, the color of DPPH^•^ solution changed from violet to yellow. As shown in Figure 10, uncoated and PEC-coated PLA mats loaded with RA exhibited DPPH^•^ scavenging ability comparable to that of the free RA. For the PLA/RA, (HA/Ch)-*coat*-PLA/RA, (Ch/HA)-*coat*-PLA/RA, [HA/(Ch/RA)]-*coat*-PLA/RA, and [(Ch/RA)/HA]-*coat*-PLA/RA mats, the absorbance of the DPPH^•^ solution decreased by 94–95%, while for RA solution it dropped by 95.1 ± 0.1%. PLA mats coated with HA/(Ch/RA) and (Ch/RA)/HA also led to a significant reduction in DPPH^•^ absorbance, by 95.2 ± 0.1% and 95.0 ± 0.1%, respectively. The obtained results are consistent with data from other studies [27,31], indicating that RA incorporated into electrospun materials retained its high antioxidant activity. In addition, RA incorporated in the PEC coating between Ch and HA exhibited high antioxidant activity, close to that of RA solution.

### 3.4. Assessment of the Antibacterial and Antifungal Activity of the Fibrous Materials

**Figure 11:**
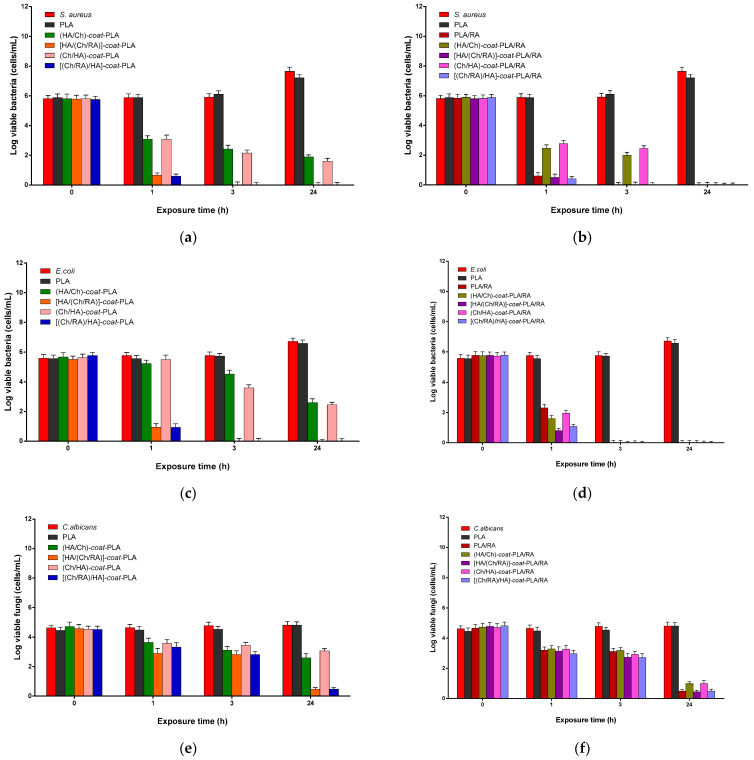
Logarithmic plot of the viable bacteria (fungi) cell number versus the exposure time for the control (bacteria (fungi) cell suspension), PLA mat, (HA/Ch)-*coat*-PLA mat, [HA/(Ch/RA)]-*coat*-PLA mat, (Ch/HA)-*coat*-PLA mat, [(Ch/RA)/HA]-*coat*-PLA mat, PLA/RA mat, (HA/Ch)-*coat*-PLA/RA mat, [HA/(Ch/RA)]-*coat*-PLA/RA mat, (Ch/HA)-*coat*-PLA/RA mat, and [(Ch/RA)/HA]-*coat*-PLA/RA mat. Tests have been carried out against (**a**,**b**) *S. aureus*, (**c**,**d**) *E. coli*, and (**e**,**f**) *C. albicans*. Data represent the mean ± standard deviation (*n* = 3). The RA concentration was 2200 μg/mL in (**a**–**d**) and 5200 μg/mL in (**e**,**f**).

The antibacterial and antifungal activity (bacteriostatic or bactericidal) of uncoated and PEC-coated PLA/RA mats, as well as [(Ch/RA)/HA]-*coat*-PLA, [HA/(Ch/RA)]-*coat*-PLA, [(Ch/RA)/HA]-*coat*-PLA/RA, and [HA/(Ch/RA)]-*coat*-PLA/RA mats, against Gram-positive bacteria *S. aureus*, Gram-negative bacteria *E. coli*, and fungi *C. albicans*, was tested in liquid medium by counting viable bacterial or fungal cells for 1, 3, and 24 h. The antibacterial and antifungal activity of uncoated and PEC-coated PLA mats was also assessed. It was found that for PLA mats, the concentration of *S. aureus* cells increased over time, and log(CFU/mL) reached 7.2 ± 0.2 at 24 h (Figure 11a). The *S. aureus* control also grew normally and log(CFU/mL) was 7.6 ± 0.3 after a 24 h contact. The coating of PLA mats with PEC Ch/HA or HA/Ch resulted in a reduction of the *S. aureus* titer to 1.6 ± 0.2 or 1.9 ± 0.1, respectively, after a contact time of 24 h, indicating that these materials exhibited bacteriostatic activity (Figure 11a). In contrast, PLA mats coated with PEC [(Ch/RA)/HA] or [HA/(Ch/RA)] (RA content—2200 μg/mL) killed all *S. aureus* bacteria within 3 h, i.e., these materials demonstrated bactericidal activity. A difference was observed in the rate of inhibition of *S. aureus* growth for PLA/RA mats and PLA/RA mats coated with PEC or PEC containing RA, at the same concentration of RA (2200 μg/mL) (Figure 11b). For PLA/RA, [HA/(Ch/RA)]-coat-PLA/RA, and [(Ch/RA)/HA]-*coat*-PLA/RA mats, the *S. aureus* titer decreased to 0.6, 0.5, and 0.4 log, respectively, after 1 h. For (HA/Ch)-*coat*-PLA/RA and (Ch/HA)-*coat*-PLA/RA mats, the titer of *S. aureus* dropped to 2.5 and 2.8 log over the same contact time. Complete inhibition of *S. aureus* bacterial growth was observed after 3 h for PLA/RA, [HA/(Ch/RA)]-*coat*-PLA/RA, and [(Ch/RA)/HA]-*coat*-PLA/RA mats, whereas for (HA/Ch)-*coat*-PLA/RA and (Ch/HA)-*coat*-PLA/RA mats it was recorded after 24 h. The observed difference in the rate of manifested antibacterial activity against *S. aureus* might be attributed to variations in the RA release profiles from RA-containing fibrous materials of different composition and design. Furthermore, the deposition sequence of Ch and HA during the dip coating of PLA/RA mats did not affect the rate of antibacterial activity.

It was found that all RA-containing mats killed all of the *E. coli* bacteria within 3 h at the same RA concentration of 2200 µg/mL. Differences in the inhibition rate of *E. coli* growth by uncoated and PEC-coated mats of various design containing RA were observed at a contact time of 1 h (Figure 11c,d). In contrast, in the case of (HA/Ch)-*coat*-PLA and (Ch/HA)-*coat*-PLA mats, only a reduction in *E. coli* titer by 2.6 and 2.45 log units was achieved, respectively, after a 24 h of contact. On the other hand, PLA mats did not affect the growth of *E. coli*, and the number of bacteria increased to 6.6 log at 24 h.

As shown in Figure 11e,f, uncoated or coated with PEC RA-containing mats exhibited weaker inhibitory activity against fungi *C. albicans* compared to that against bacteria *S. aureus* and *E. coli*. At an RA concentration of 5200 μg/mL, the number of the fungal cells decreased to 0.50, 0.45, 0.50, 0.46, and 0.47 log, respectively, after a 24 h contact of *C. albicans* with PLA/RA, [HA/(Ch/RA)]-*coat*-PLA/RA, [(Ch/RA)/HA]-*coat*-PLA/RA, [HA/(Ch/RA)]-*coat*-PLA, and [(Ch/RA)/HA]-*coat*-PLA mats. In the case of PLA/RA mats coated with PEC (Ch/HA or HA/Ch), the fungal titer decreased to approximately 1.0 log over the same time period. On the other hand, PEC-coated mats that do not contain RA resulted in only a slight reduction in the number of viable fungal cells. For example, after 24 h of incubation, the cell titer was dropped to only 2.6 and 3.06 log for (HA/Ch)-*coat*-PLA and (Ch/HA)-*coat*-PLA mats, respectively. In contrast, for neat PLA mats, the number of viable fungal cells after contact of 24 h reached 4.81 log, which is close to that of the *C. albicans* control.

Therefore, the results of the conducted microbiological tests indicated that the rate of the exhibited antibacterial and antifungal activity did not depend on the deposition sequence of Ch and HA during the dip coating of PLA/RA mats, but rather on the composition and design of the uncoated and PEC-coated fibrous materials containing RA, which demonstrated strong antibacterial and antifungal activity.

In the present study, the behavior of PLA fibrous materials, as well as uncoated and [HA/(Ch/RA)]- or [(Ch/RA)/HA]-coated PLA/RA fibrous materials, in contact with the pathogenic bacteria *S. aureus*, was investigated. The adhesion of *S. aureus* cells onto the surface of the studied mats was examined by SEM. As shown in Figure S14a (Supplementary Materials), a large number of bacteria adhere to the surface of PLA. This is consistent with data from our previous studies [9] and might be attributed to the hydrophobic nature of the surface. In contrast, the incorporation of RA into the bulk of PLA fibers caused an inhibition of the growth of pathogenic *S. aureus* bacteria on the fiber surface (Supplementary Materials, Figure S14b). A similar trend for preventing the *S. aureus* adhesion and their proliferation was also detected for fibrous materials containing RA both in the fiber bulk and in the PEC coating (Supplementary Materials, Figure S14c,d). Because of the biocidal activity and anti-adhesive properties, these materials are promising candidates for a number of applications in the biomedical field.

Overall, the performed in vitro antibacterial and antifungal assays revealed the considerable efficacy of the prepared PEC-coated fibrous materials loaded with RA against pathogenic bacteria and fungi. Nevertheless, these studies cannot fully replicate the complex wound environment, including immune responses, tissue interactions, blood supply, and biofilm formation. Therefore, future studies should focus on performing in vivo animal tests to evaluate cytotoxicity, tissue regeneration, inflammation, and overall wound healing performance under physiological conditions. Such studies are crucial for enabling the clinical application of the developed PEC-coated fibrous materials loaded with the bioactive compound RA.

## 4. Conclusions

A hierarchical design strategy for electrospun PLA-based fibrous materials with tunable functionality was demonstrated through the integration of bulk loading and surface engineering via chitosan/hyaluronic acid polyelectrolyte complexes (PECs). This study establishes that both the architecture of the PEC coating and the spatial localization of rosmarinic acid (RA)—within the fiber bulk, the coating, or both—govern the release behavior and biological performance of the system.

The PEC layer was shown to play a dual functional role, acting either as a diffusion barrier that retards RA release or as a surface-associated reservoir enabling rapid initial release, depending on the material design, which was a prerequisite for achieving a sustained or fast biological effect. This also provides a versatile mechanism for tuning release profiles without altering the fiber bulk composition. Clear structure–property–function relationships were identified, linking coating configuration and RA distribution to hydrophilicity, mechanical performance, and bioactivity. The developed PEC-coated materials exhibited enhanced wettability and mechanical properties. Additionally, the mechanical properties of the materials were within the range characteristic of human skin, indicating their applicability, particularly in the field of wound healing. The mats loaded with RA, whether PEC-coated or uncoated, demonstrated high antioxidant activity (ca. 94–95%) close to that of the neat RA. It was found that the rate of the antibacterial and antifungal activity of the obtained fibrous materials depended mainly on the design and composition of the non-woven textile. The prepared RA-containing fibrous materials displayed bactericidal activity against pathogenic bacteria *S. aureus* and *E. coli*. The neat and RA-loaded mats coated with PEC containing RA led to a decrease of the *C. albicans* titer up to ca. 0.5 log after a 24 h exposure, while at the same contact time the PEC-coated mats loaded with RA resulted in a diminishing of the *C. albicans* titer by 1.0 log units. The incorporation of RA into the bulk of the fibers or into the PEC coating resulted in inhibition of *S. aureus* growth on the fiber surface. The proposed approach advances the rational design of multifunctional fibrous biomaterials and offers broad potential for applications in wound healing and controlled drug delivery.

## Data Availability

The data that support the findings of this study are available from the corresponding author upon request.

## Supplementary Materials

The following supporting information can be downloaded at https://www.mdpi.com/article/10.3390/polym18101207/s1, Scheme S1: Schematic representation of the cross-section of a fiber from PLA or PLA/RA; Figure S1: CLSM images of PLA/RA (10 wt%) fibers. PLA/RA fibers are stained in blue by the embedded RA. Representative images of Z-layers at a depth of 4 µm (a), 5 µm (b), 6 µm (c) and 7 µm (d). The Z step of the images is 1 μm. Scale bars = 10 µm; Figure S2: Confocal laser scanning microscopy (CLSM) images of fibers from [(Ch/RA)/(HA/RhB)]-*coat*-PLA/RA. [(Ch/RA)/(HA/RhB)]-*coat*-PLA/RA fibers are stained in red by the embedded RhB, and in blue by the embedded RA. Representative images of Z-layers at a depth of 2 µm, 3 µm, 4 µm, 5 µm, 6 µm and 7 µm are shown. The Z step of the images is 1 μm. Scale bars = 5 µm; Figure S3: Confocal laser scanning microscopy (CLSM) images of fibers from [(HA/RhB)/(Ch/RA)]-*coat*-PLA. [(HA/RhB)/(Ch/RA)]-*coat*-PLA fibers are stained in blue by the embedded RA, and in red by the embedded RhB. Representative images of Z-layers at a depth of 3 µm, 4 µm, 5 µm, 6 µm, and 7 µm are shown. The Z step of the images is 1 μm. Scale bars = 5 µm; Figure S4: Confocal laser scanning microscopy (CLSM) images of fibers from [(HA/RhB)/(Ch/RA)]-*coat*-PLA/RA. [(HA/RhB)/(Ch/RA)]-*coat*-PLA/RA fibers are stained in blue by the embedded RA, and in red by the embedded RhB. Representative images of Z-layers at a depth of 2 µm, 3 µm, 4 µm, and 5 µm are shown. The Z step of the images is 1 μm. Scale bars = 5 µm; Figure S5: ATR–FTIR spectra of: (a) Ch, (b) HA and (c) RA; Figure S6: ATR–FTIR spectra of mats: (a) (Ch/HA)-*coat*-PLA, (b) [(Ch/RA)/HA]-*coat*-PLA, (c) (Ch/HA)-*coat*-PLA/RA and (d) [(Ch/RA)/HA]-*coat*-PLA/RA; Figure S7: XPS peak fittings for PLA mat [(a) C_1s_, (b) O_1s_], (HA/Ch)-*coat*-PLA mat [(c) C_1s_, (d) O_1s_, (e) N_1s_, (f) Na_1s_] and [HA/(Ch/RA)]-*coat*-PLA mat [(g) C_1s_, (h) O_1s_, (i) N_1s_, (j) Na_1s_]; Figure S8: XPS peak fittings for (Ch/HA)-*coat*-PLA mat [(a) C_1s_, (b) O_1s_, (c) N_1s_, (d) Na_1s_] and [(Ch/RA)/HA]-*coat*-PLA mat [(e) C_1s_, (f) O_1s_, (g) N_1s_, (h) Na_1s_]; Figure S9: XPS peak fittings for (Ch/HA)-*coat*-PLA/RA mat [(a) C_1s_, (b) O_1s_, (c) N_1s_, (d) Na_1s_] and [(Ch/RA)/HA]-*coat*-PLA/RA mat [(e) C_1s_, (f) O_1s_, (g) N_1s_, (h) Na_1s_]; Figure S10: XRD patterns of: (A) (a) PLA, (b) (Ch/HA)-*coat*-PLA, (c) (HA/Ch)-*coat*-PLA, (d) [(Ch/RA)/HA]-*coat*-PLA, (e) [HA/(Ch/RA)]-*coat*-PLA; (B) (a) PLA/RA(10 wt%), (b) (Ch/HA)-*coat*-PLA/RA, (c) (HA/Ch)-*coat*-PLA/RA, (d) [(Ch/RA)/HA]-*coat*-PLA/RA, (e) [HA/(Ch/RA)]-*coat*-PLA/RA and (C) RA powder; Figure S11: TGA thermograms of PEC-coated PLA (a) or PLA/RA (b) mats; Figure S12: Stress–strain curves of PLA/RA-based fibrous materials; Figure S13: Release profiles of RA from mats: [(Ch/RA)/HA]-*coat*-PLA and [HA/(Ch/RA)]-*coat*-PLA in PBS (pH 7.4, I = 0.1); 37 °C; Figure S14: SEM micrographs of fibrous materials that have been incubated in *S. aureus* cell culture (10^7^ cells/mL) for 24 h at 37 °C, (a) PLA, (b) PLA/RA, (c) [HA/(Ch/RA)]-*coat*-PLA/RA and (d) [(Ch/RA)/HA]-*coat*-PLA/RA; magnification ×6000; Table S1: Dynamic viscosity (η) and conductivity (σ) of the spinning solutions, and mean fiber diameter of the electrospun mats; Table S2: Mean fiber diameter of the PEC-coated electrospun mats; Table S3: Tensile characteristics of the fibrous materials.
