# Supplementary material for: Surface Engineering of Electrospun PLA Fibers via Chitosan/Hyaluronic Acid Polyelectrolyte Complexes for Tunable Release of Rosmarinic Acid
Source: Polymers (Basel). 2026 May 15;18(10):1207. doi: 10.3390/polym18101207 (PMC13210676; doi:10.3390/polym18101207)
Supplement: Supplementary file 1 [file polymers-18-01207-s001.zip › polymers-4305261-supplementary.pdf]

Supplementary Materials

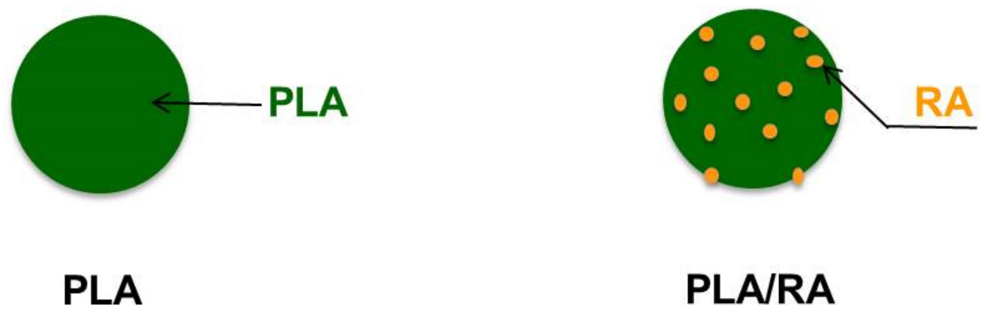

Scheme S1. Schematic representation of the cross-section of a fiber from PLA or PLA/RA.

Table S1. Dynamic viscosity (η) and conductivity (σ) of the spinning solutions, and mean fiber diameter of the electrospun mats.

Electrospun mats	η (cP)	σ (μS/cm)	d (nm)
PLA	400	5.1	730 ± 220
PLA/RA(5%)	270	5.7	660 ± 115
PLA/RA(10%)	215	5.8	620 ± 130

Table S2. Mean fiber diameter of the PEC-coated electrospun mats.

Electrospun mats	d (nm)
**PLA-based mats**	
(Ch/HA)-*coat*-PLA	940 ± 315
[(Ch/RA)/HA]-*coat*-PLA	900 ± 260
(HA/Ch)-*coat*-PLA	805 ± 260
[HA/(Ch/RA)]-*coat*-PLA	820 ±200
**PLA/RA-based mat**	
(Ch/HA)-*coat*-PLA/RA	785 ± 145
[(Ch/RA)/HA]-*coat*-PLA/RA	760 ± 165
(HA/Ch)-*coat*-PLA/RA	730 ± 205
[HA/(Ch/RA)]-*coat*-PLA/RA	780 ± 155

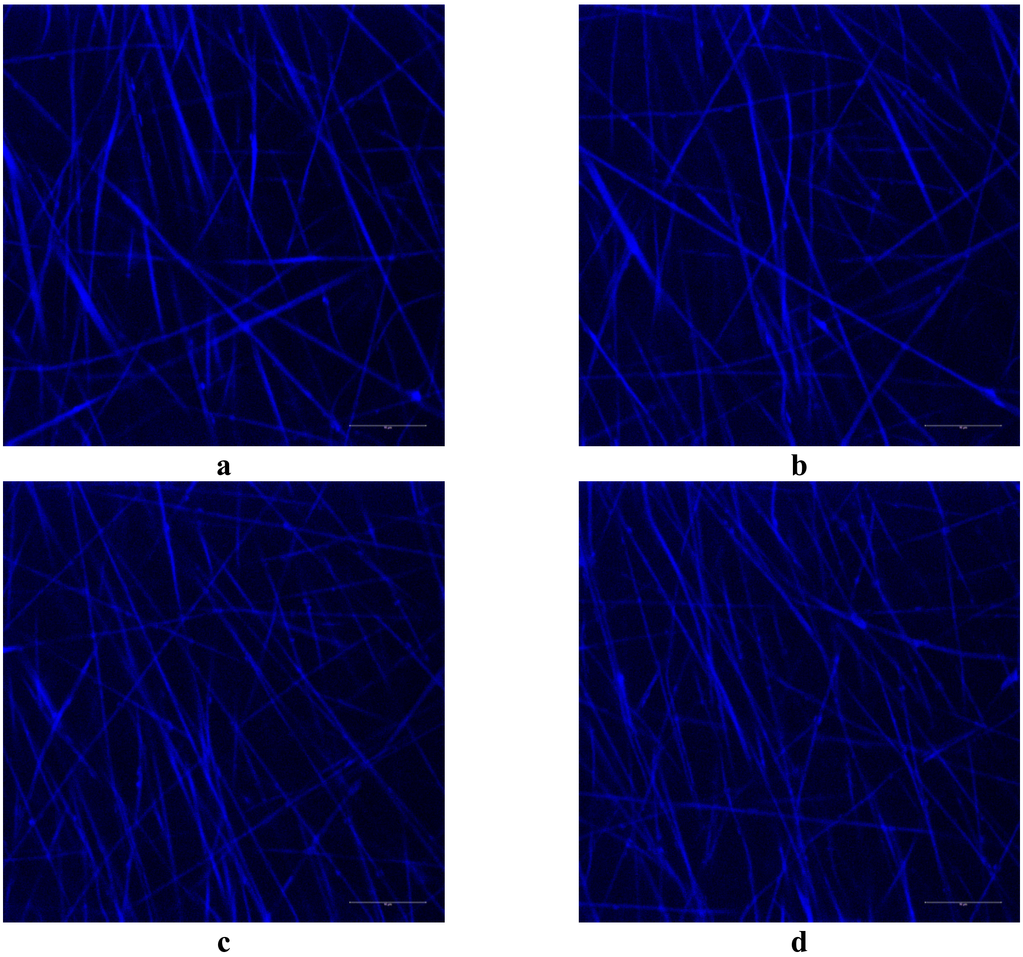

Figure S1. CLSM images of PLA/RA (10 wt%) fibers. PLA/RA fibers are stained in blue by the embedded RA. Representative images of Z-layers at a depth of 4 µm (a), 5 µm (b), 6 µm (c) and 7 µm (d). The Z step of the images is 1 µm. Scale bars = 10 µm.

Z layer | stained red | merged image | stained blue

Z at a depth of 2 μm

Z at a depth of 3 μm

Z at a depth of 4 μm

Z at a depth of 5 μm

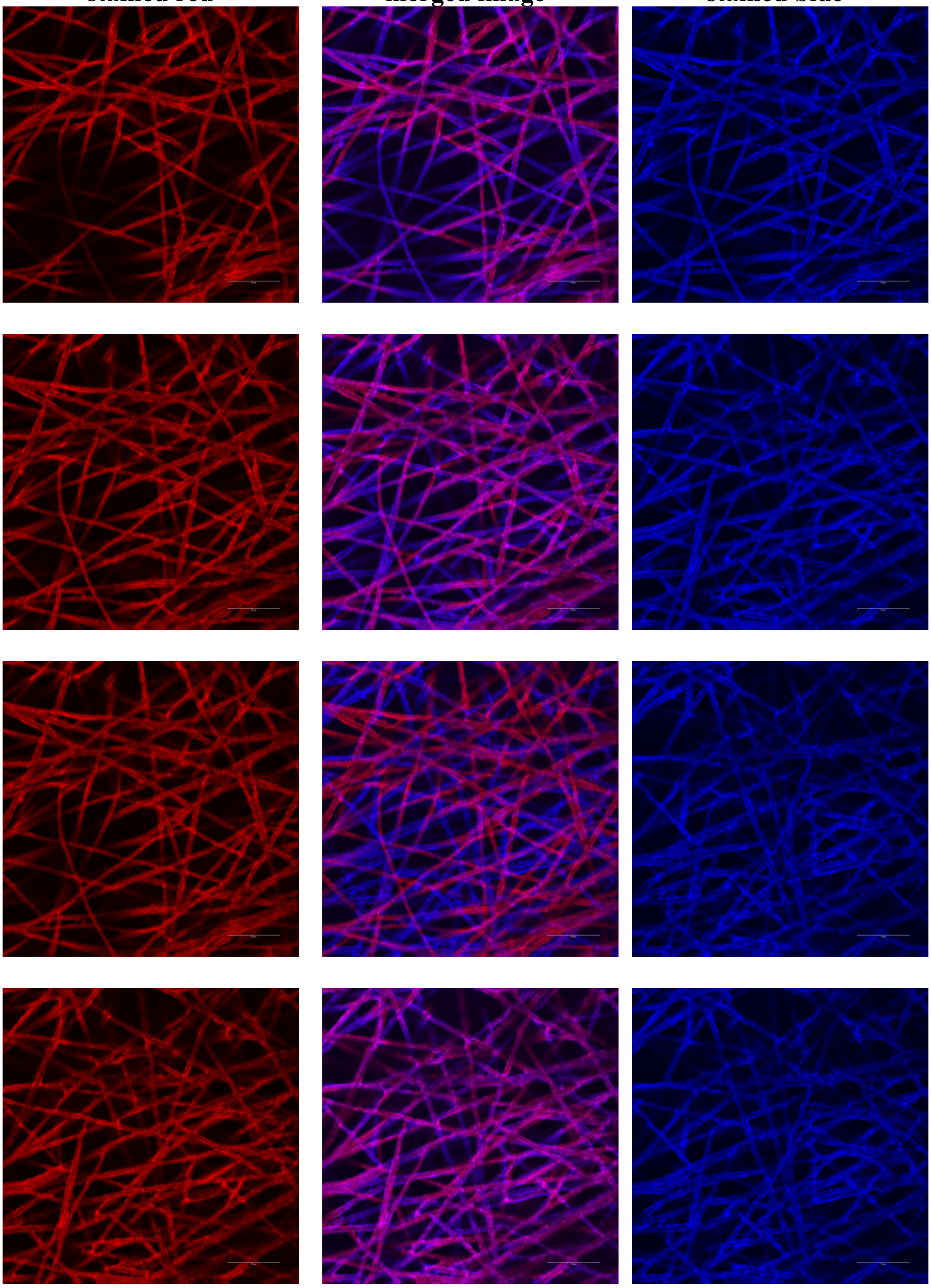

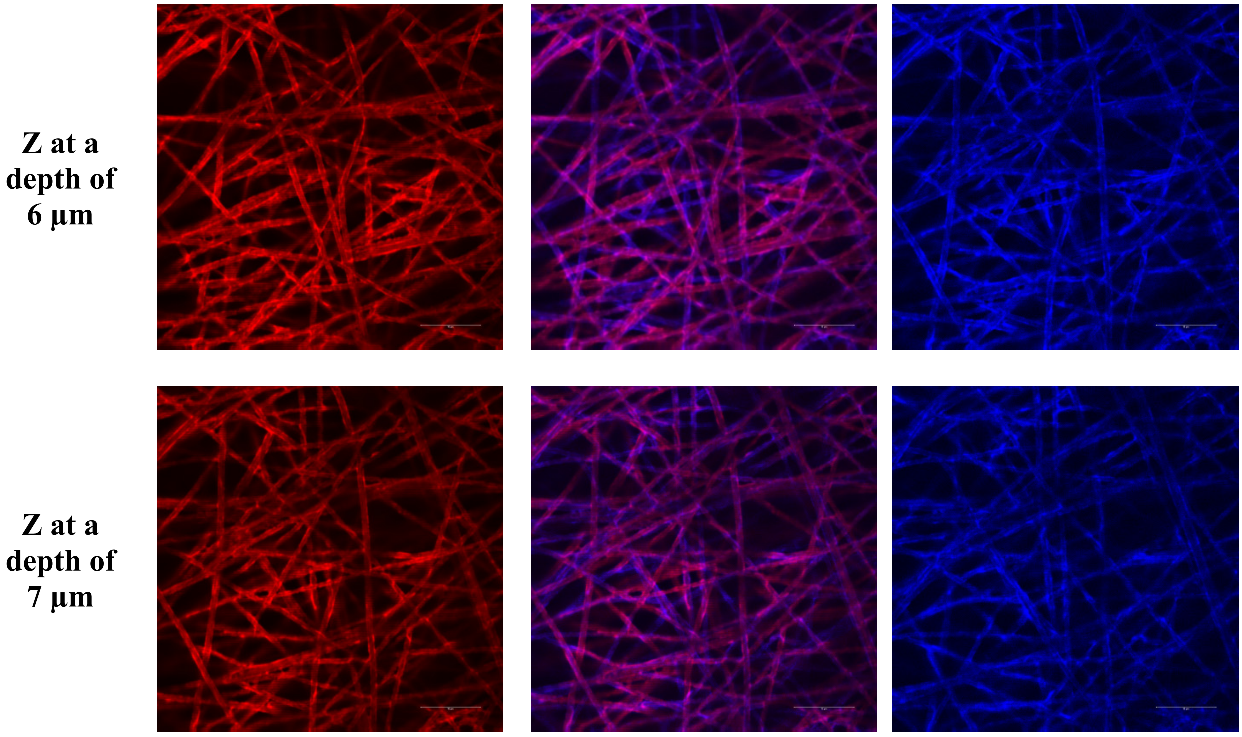

Figure S2. Confocal laser scanning microscopy (CLSM) images of fibers from [(Ch/RA)/(HA/RhB)]-*coat*-PLA/RA. [(Ch/RA)/(HA/RhB)]-*coat*-PLA/RA fibers are stained in red by the embedded RhB, and in blue by the embedded RA. Representative images of Z-layers at a depth of 2 µm, 3 µm, 4 µm, 5 µm, 6 µm and 7 µm are shown. The Z step of the images is 1 µm. Scale bars = 5 µm.

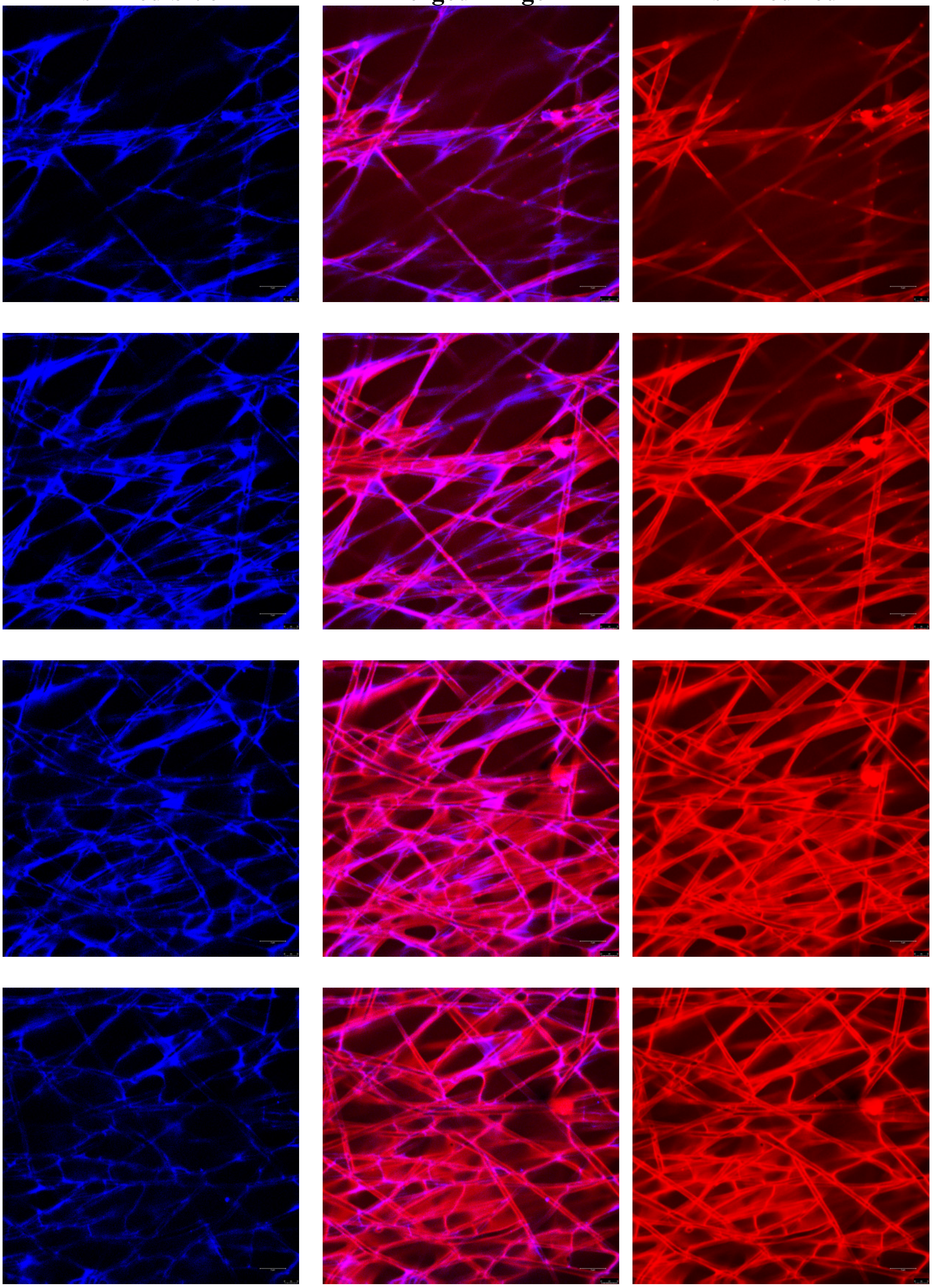
Z layer
stained blue
merged image
stained red
Z at a depth of 3 µm
Z at a depth of 4 µm
Z at a depth of 5 µm
Z at a depth of 6 µm

Z at a depth of 7 μm

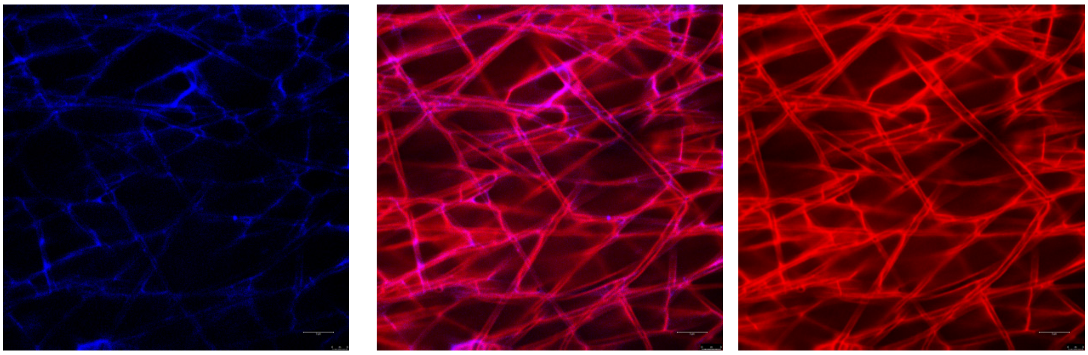

Figure S3. Confocal laser scanning microscopy (CLSM) images of fibers from [(HA/RhB)/(Ch/RA)]-*coat*-PLA. [(HA/RhB)/(Ch/RA)]-*coat*-PLA fibers are stained in blue by the embedded RA, and in red by the embedded RhB. Representative images of Z-layers at a depth of 3 μm, 4 μm, 5 μm, 6 μm, and 7 μm are shown. The Z step of the images is 1 μm. Scale bars = 5 μm.

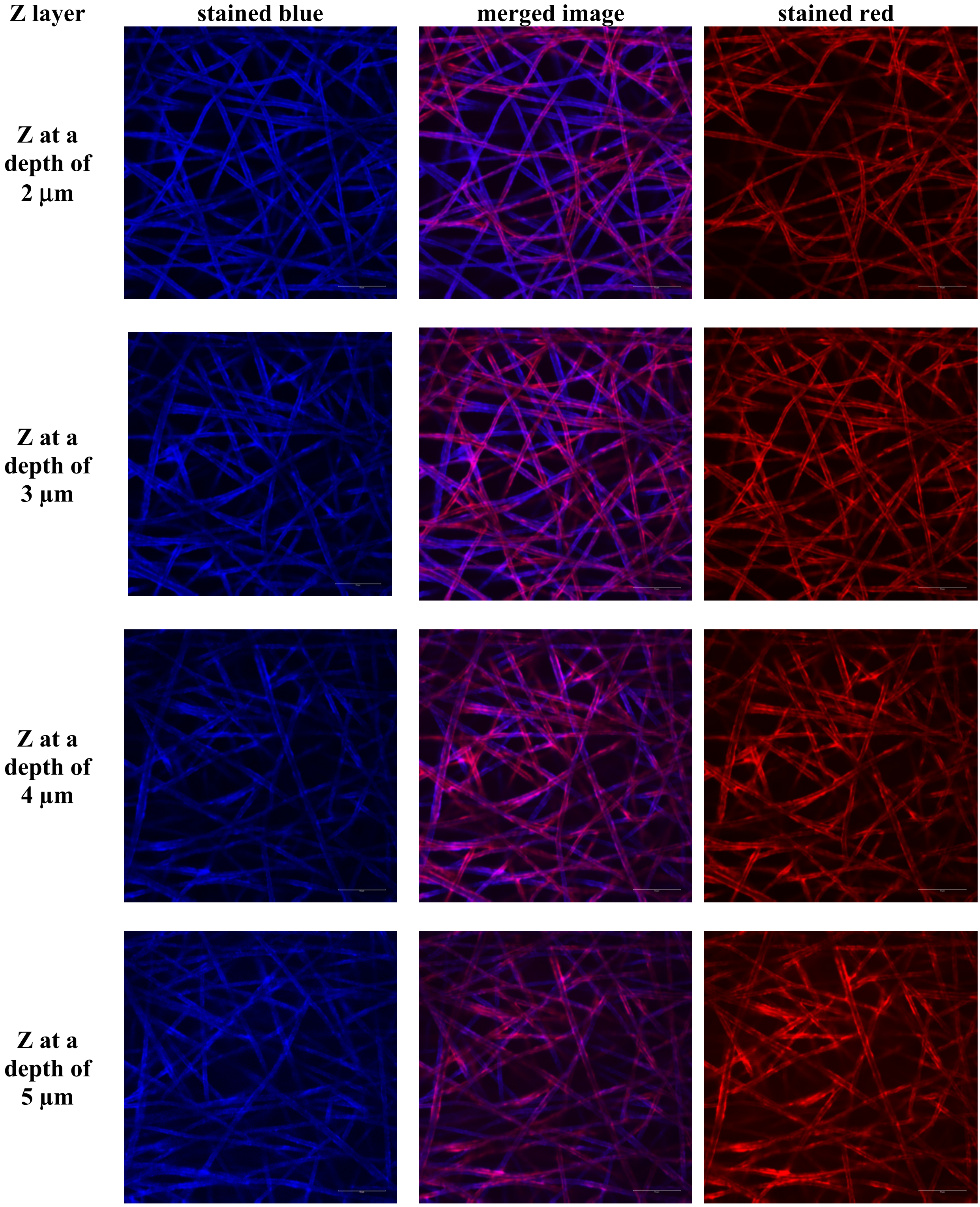

Figure S4. Confocal laser scanning microscopy (CLSM) images of fibers from [(HA/RhB)/(Ch/RA)]-*coat*-PLA/RA. [(HA/RhB)/(Ch/RA)]-*coat*-PLA/RA fibers are stained in

blue by the embedded RA, and in red by the embedded RhB. Representative images of Z-layers at a depth of 2 μm, 3 μm, 4 μm, and 5 μm are shown. The Z step of the images is 1 μm. Scale bars = 5 μm.

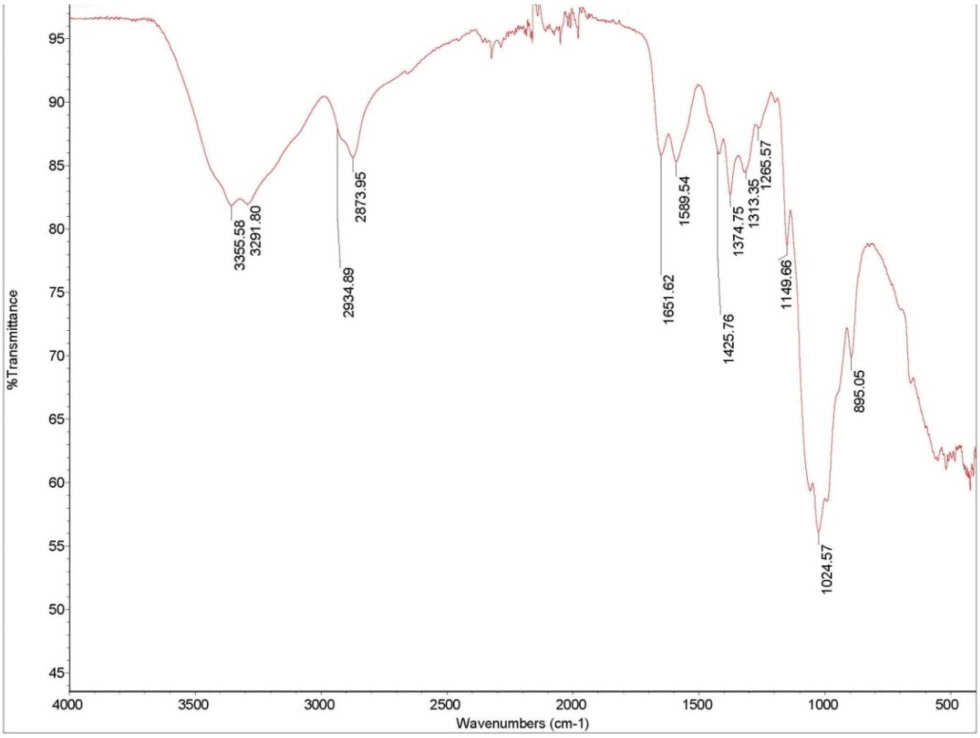
%Transmittance
Wavenumbers (cm-1)
95
90
85
80
75
70
65
60
55
50
45
4000
3500
3000
2500
2000
1500
1000
500
3355.58
3291.80
2934.89
2873.95
1651.62
1589.54
1425.76
1374.75
1313.35
1265.57
1149.66
895.05
1024.57

a

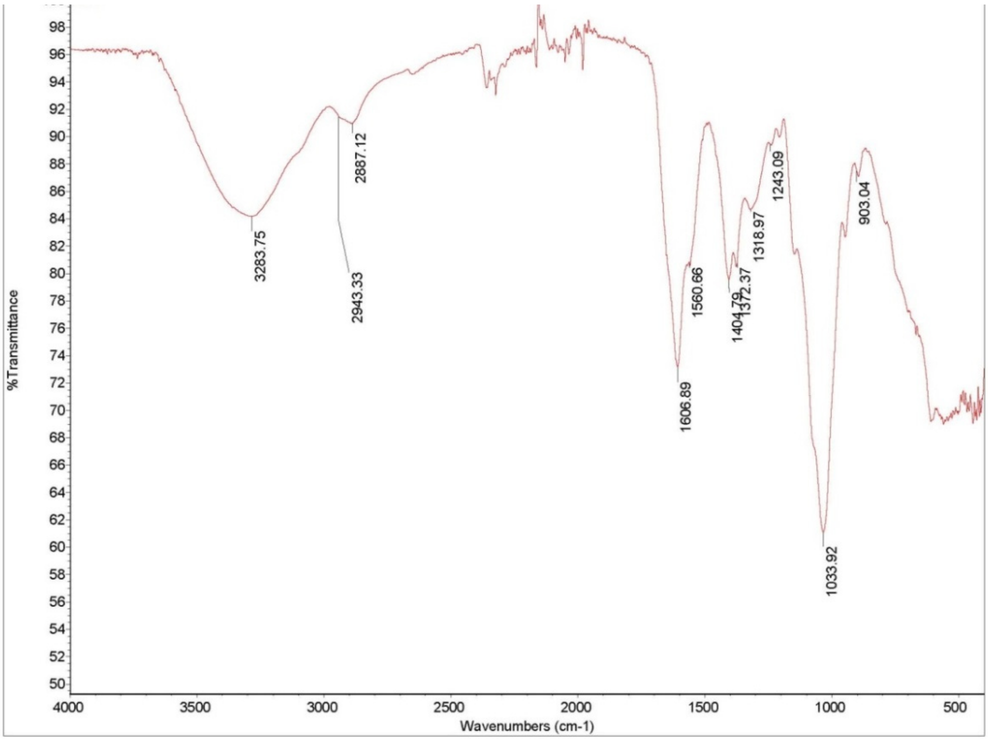
%Transmittance
Wavenumbers (cm-1)
98
96
94
92
90
88
86
84
82
80
78
76
74
72
70
68
66
64
62
60
58
56
54
52
50
4000
3500
3000
2500
2000
1500
1000
500
3283.75
2943.33
2887.12
1606.89
1560.66
1404.79
1372.37
1318.97
1243.09
903.04
1033.92

b

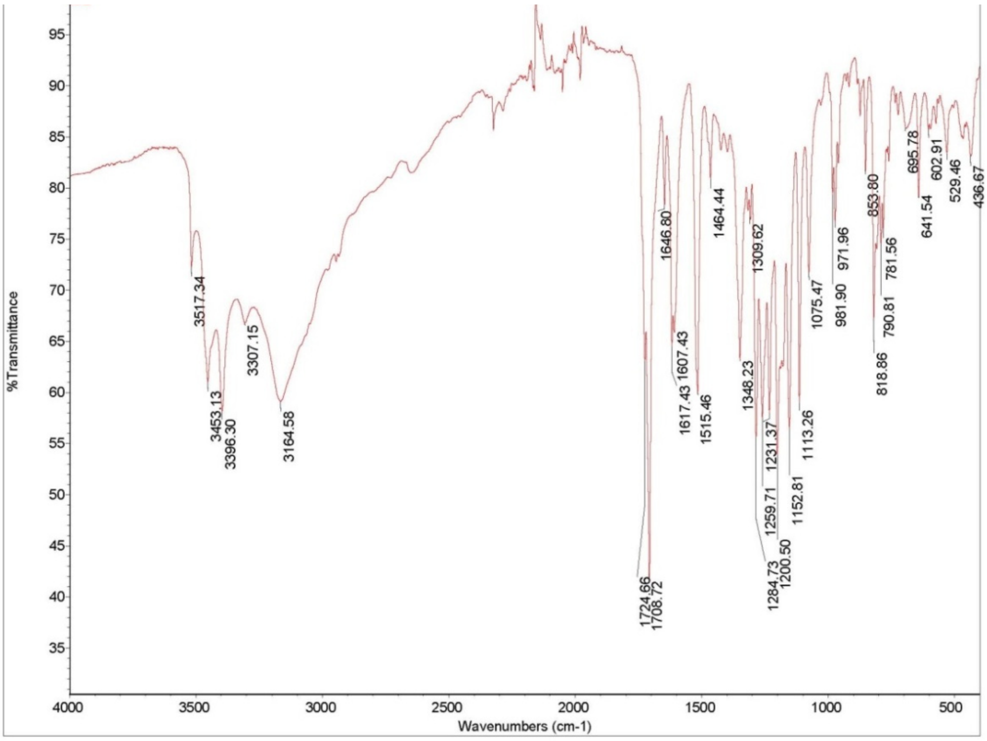

c

Figure S5. ATR–FTIR spectra of: (a) Ch, (b) HA and (c) RA.

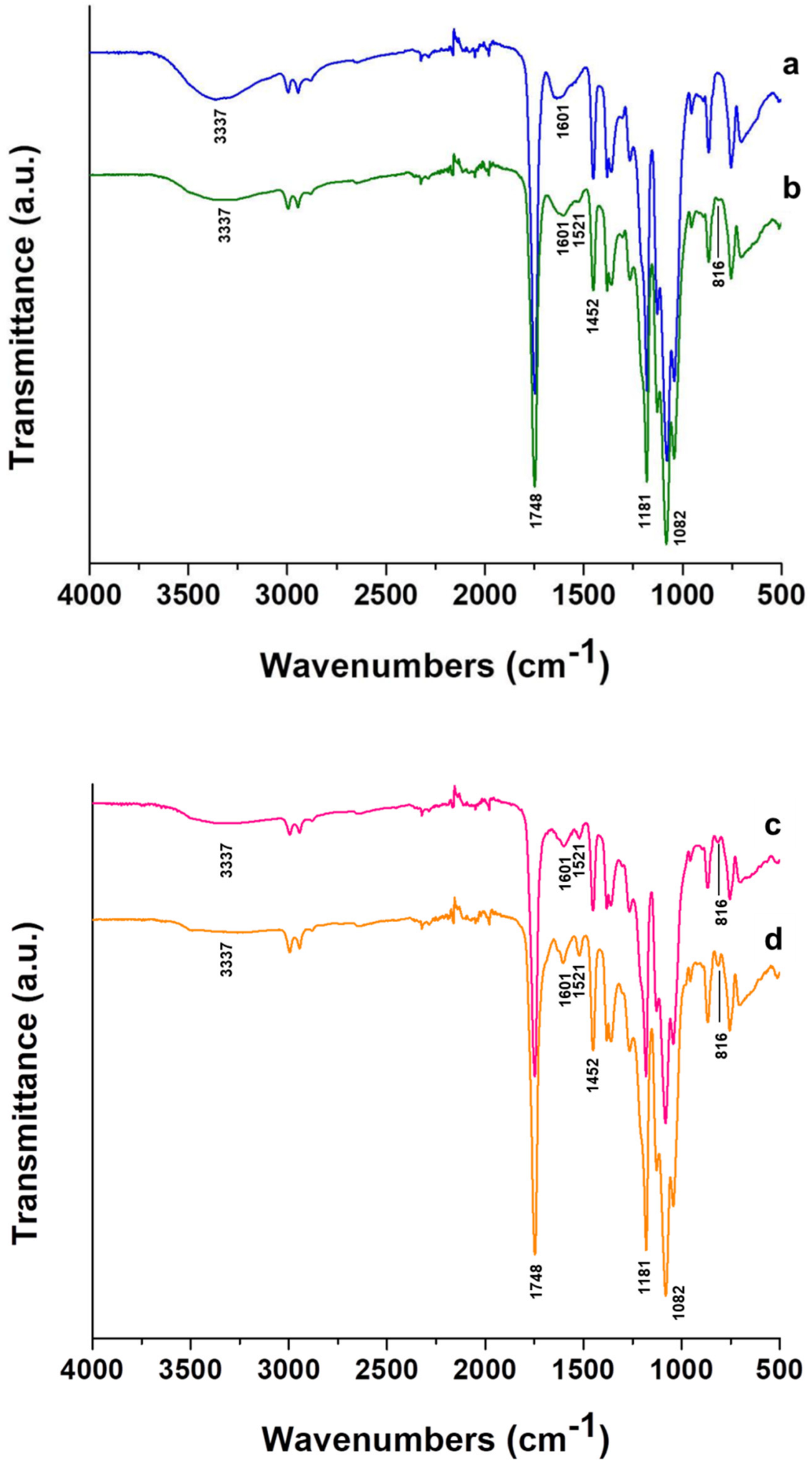

Figure S6. ATR–FTIR spectra of mats: (**a**) (Ch/HA)-*coat*-PLA, (b) [(Ch/RA)/HA]-*coat*-PLA, (c) (Ch/HA)-*coat*-PLA/RA and (d) [(Ch/RA)/HA]-*coat*-PLA/RA.

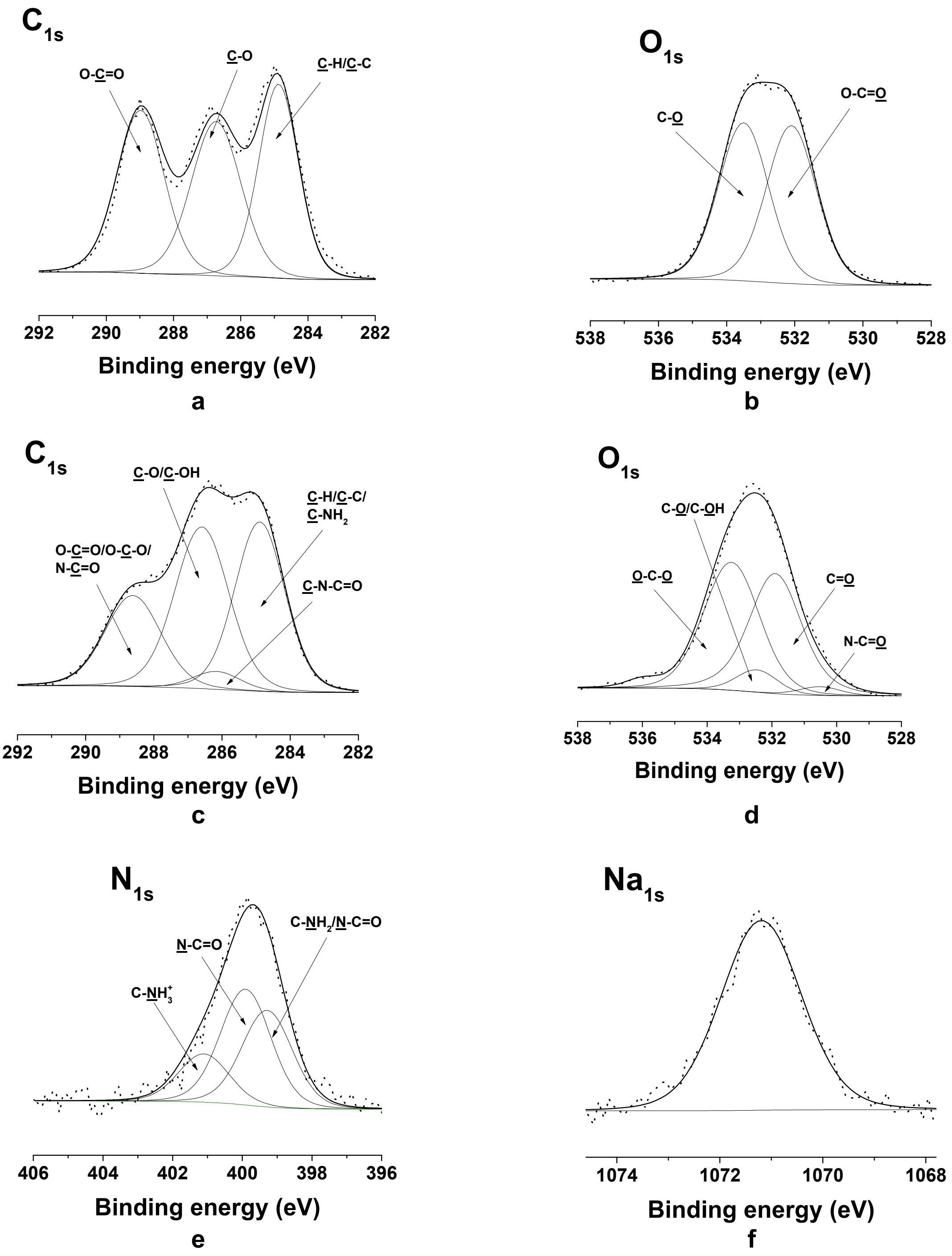
C_{1s}
O-C=O
C-O
C-H/C-C
292 290 288 286 284 282
Binding energy (eV)
a
O_{1s}
C-O
O-C=O
538 536 534 532 530 528
Binding energy (eV)
b
C_{1s}
C-O/C-OH
C-H/C-C/
$C-NH_2$
O-C=O/O-C-O/
N-C=O
C-N-C=O
292 290 288 286 284 282
Binding energy (eV)
c
O_{1s}
C-O/C-OH
O-C-O
C=O
N-C=O
538 536 534 532 530 528
Binding energy (eV)
d
N_{1s}
N-C=O
$C-NH_2$/N-C=O
$C-NH_3^+$
406 404 402 400 398 396
Binding energy (eV)
e
Na_{1s}
1074 1072 1070 1068
Binding energy (eV)
f

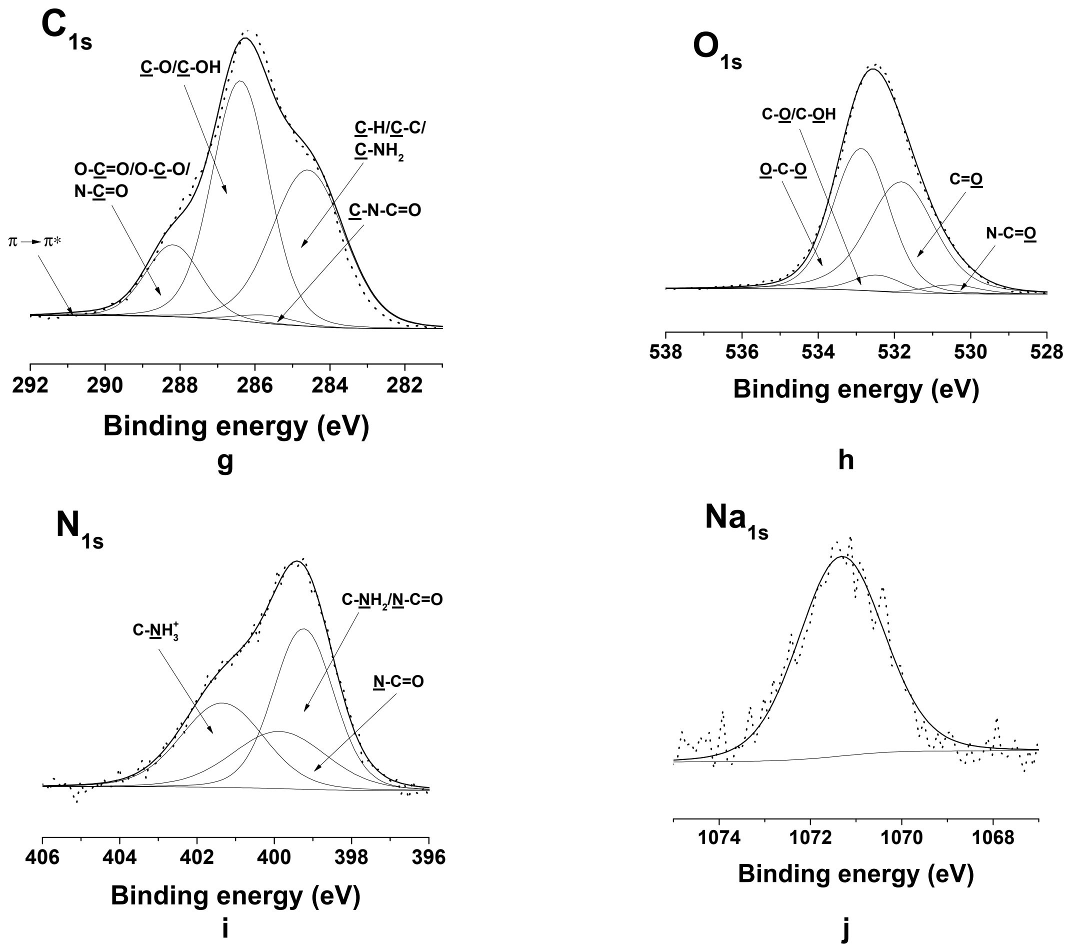

Figure S7. XPS peak fittings for PLA mat [(a) C_{1s}, (b) O_{1s}], (HA/Ch)-*coat*-PLA mat [(c) C_{1s}, (d) O_{1s}, (e) N_{1s}, (f) Na_{1s}] and [HA/(Ch/RA)]-*coat*-PLA mat [(g) C_{1s}, (h) O_{1s}, (i) N_{1s}, (j) Na_{1s}].

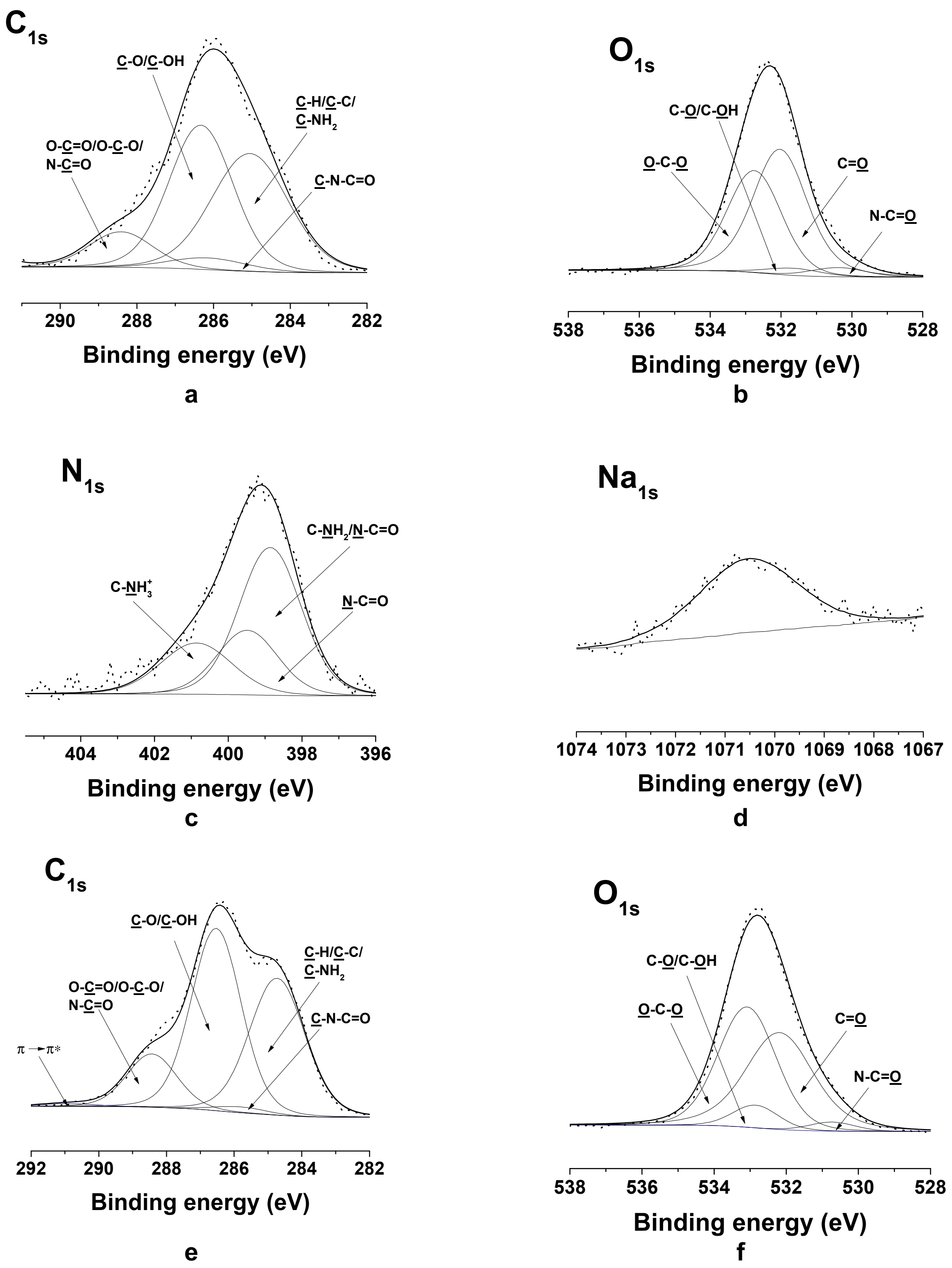
C_{1s}
C-O/C-OH
C-H/C-C/
C-NH_2
O-C=O/O-C-O/
N-C=O
C-N-C=O
290 288 286 284 282
Binding energy (eV)
a
O_{1s}
C-O/C-OH
O-C-O
C=O
N-C=O
538 536 534 532 530 528
Binding energy (eV)
b
N_{1s}
C-NH_2/N-C=O
C-NH_3^+
N-C=O
404 402 400 398 396
Binding energy (eV)
c
Na_{1s}
1074 1073 1072 1071 1070 1069 1068 1067
Binding energy (eV)
d
C_{1s}
C-O/C-OH
C-H/C-C/
C-NH_2
O-C=O/O-C-O/
N-C=O
C-N-C=O
$\pi \rightarrow \pi^*$
292 290 288 286 284 282
Binding energy (eV)
e
O_{1s}
C-O/C-OH
O-C-O
C=O
N-C=O
538 536 534 532 530 528
Binding energy (eV)
f

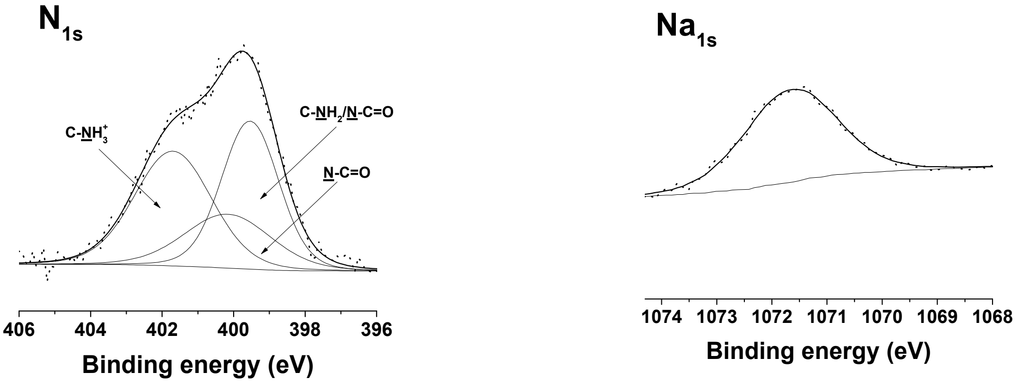

g h

Figure S8. XPS peak fittings for (Ch/HA)-*coat*-PLA mat [(a) C_{1s}, (b) O_{1s}, (c) N_{1s}, (d) Na_{1s}] and [(Ch/RA)/HA]-*coat*-PLA mat [(e) C_{1s}, (f) O_{1s}, (g) N_{1s}, (h) Na_{1s}].

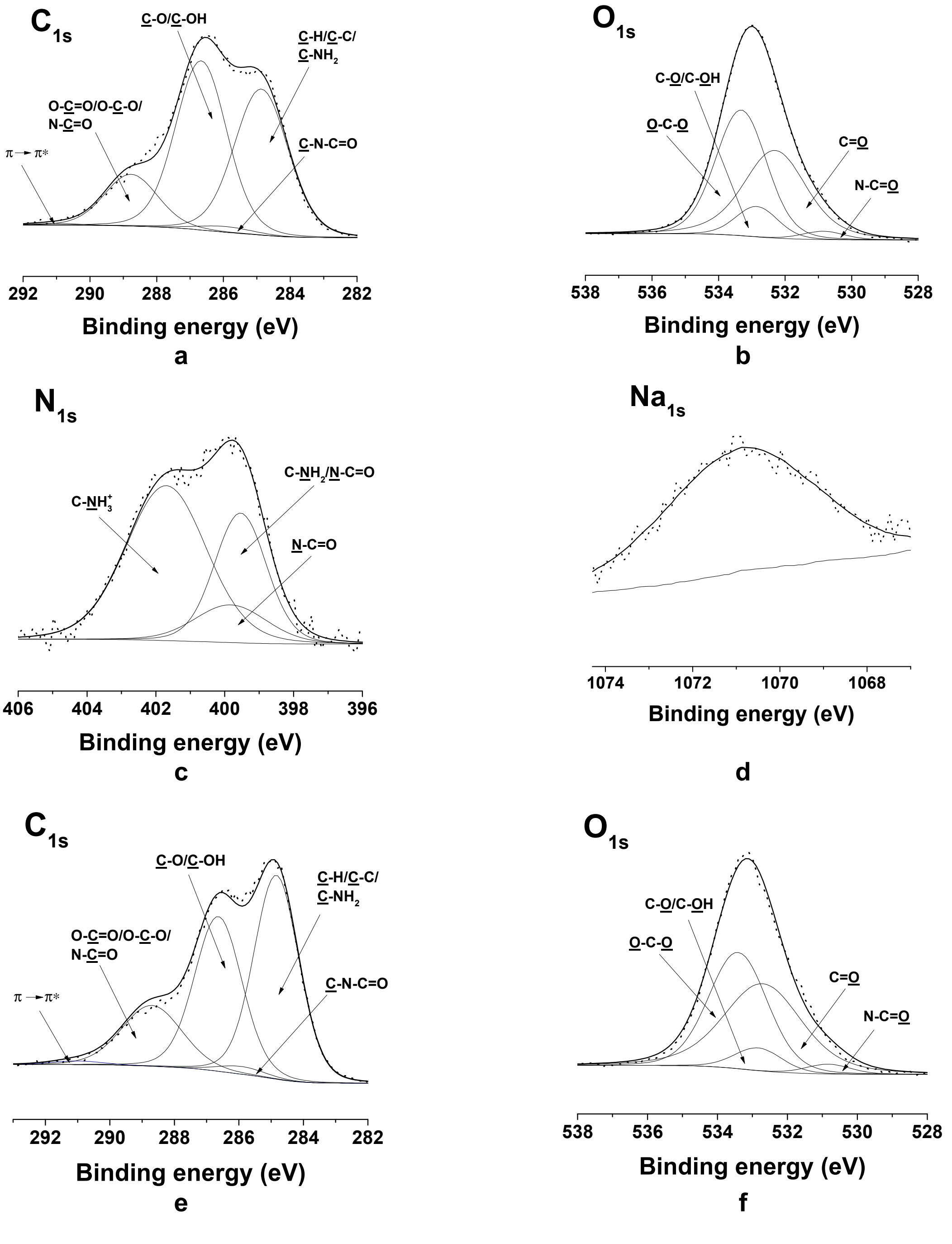
C1s
C-O/C-OH
C-H/C-C/
C-NH2
O-C=O/O-C-O/
N-C=O
π → π*
C-N-C=O
Binding energy (eV)
a
O1s
C-O/C-OH
O-C-O
C=O
N-C=O
Binding energy (eV)
b
N1s
C-NH3+
C-NH2/N-C=O
N-C=O
Binding energy (eV)
c
Na1s
Binding energy (eV)
d
C1s
C-O/C-OH
C-H/C-C/
C-NH2
O-C=O/O-C-O/
N-C=O
π → π*
C-N-C=O
Binding energy (eV)
e
O1s
C-O/C-OH
O-C-O
C=O
N-C=O
Binding energy (eV)
f

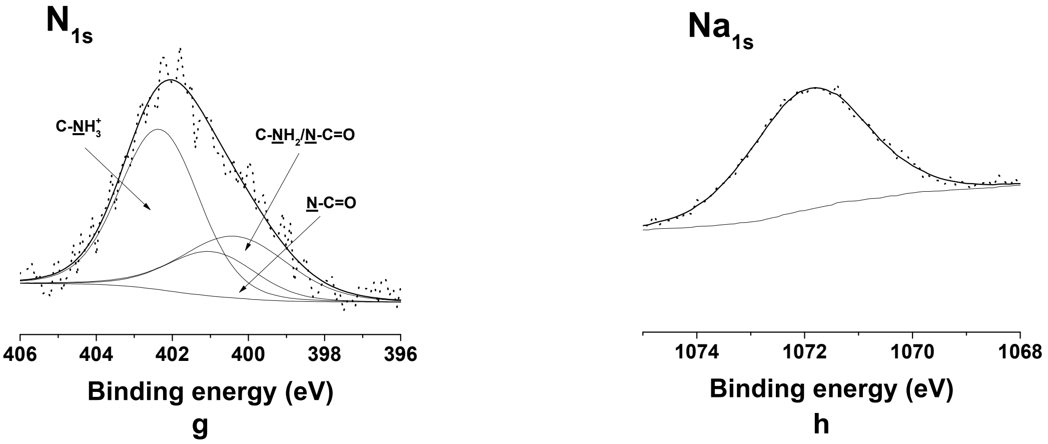

Figure S9. XPS peak fittings for (Ch/HA)-*coat*-PLA/RA mat [(a) C_{1s}, (b) O_{1s}, (c) N_{1s}, (d) Na_{1s}] and [(Ch/RA)/HA]-*coat*-PLA/RA mat [(e) C_{1s}, (f) O_{1s}, (g) N_{1s}, (h) Na_{1s}].

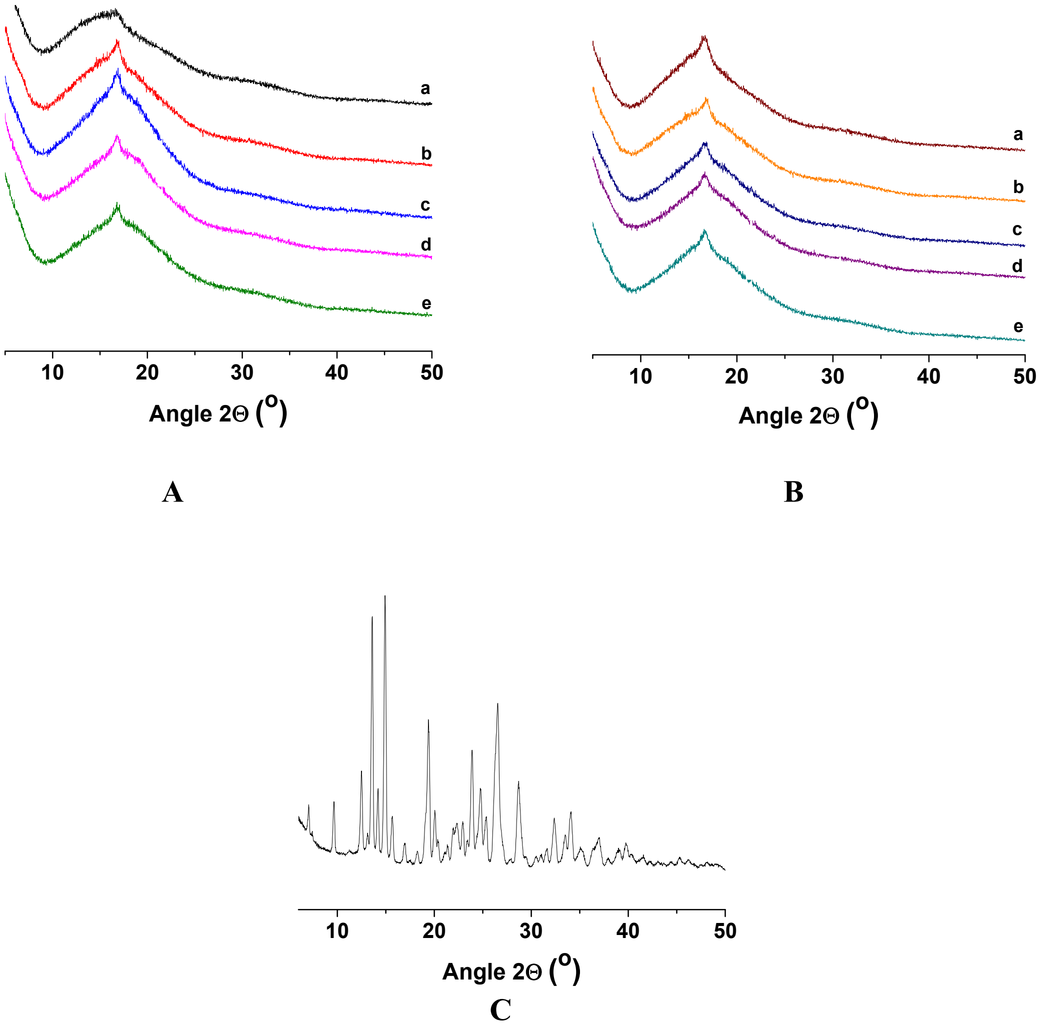

Figure S10. XRD patterns of: (A) (a) PLA, (b) (Ch/HA)-*coat*-PLA, (c) (HA/Ch)-*coat*-PLA, (d) [(Ch/RA)/HA]-*coat*-PLA, (e) [HA/(Ch/RA)]-*coat*-PLA; (B) (a) PLA/RA(10 wt%), (b) (Ch/HA)-*coat*-PLA/RA, (c) (HA/Ch)-*coat*-PLA/RA, (d) [(Ch/RA)/HA]-*coat*-PLA/RA, (e) [HA/(Ch/RA)]-*coat*-PLA/RA and (C) RA powder.

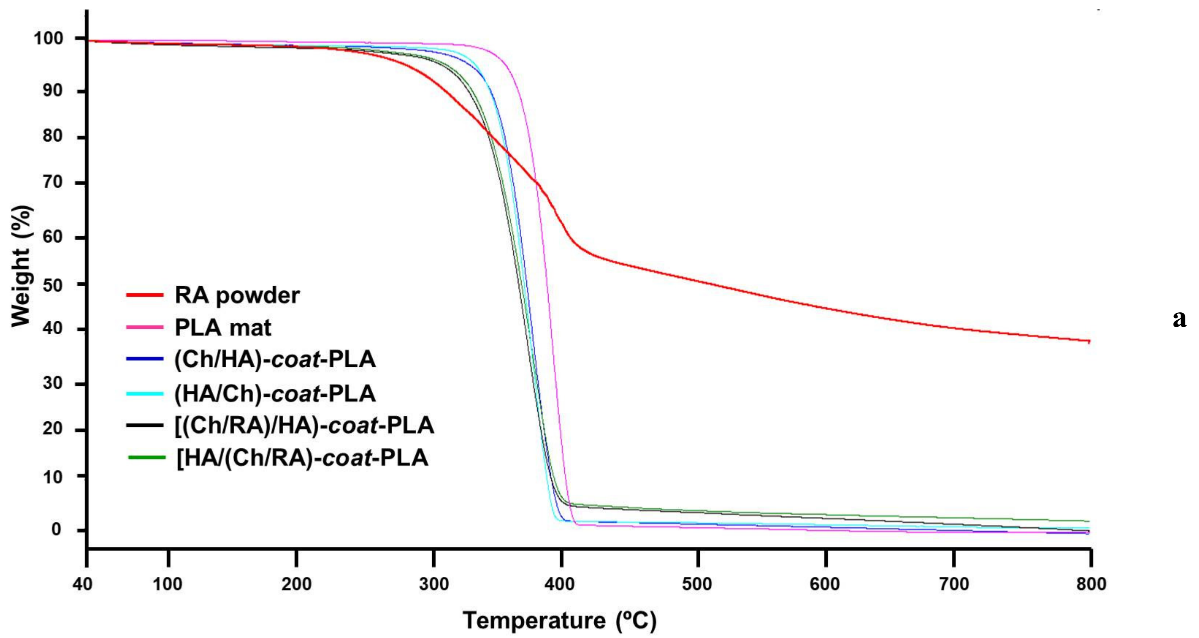

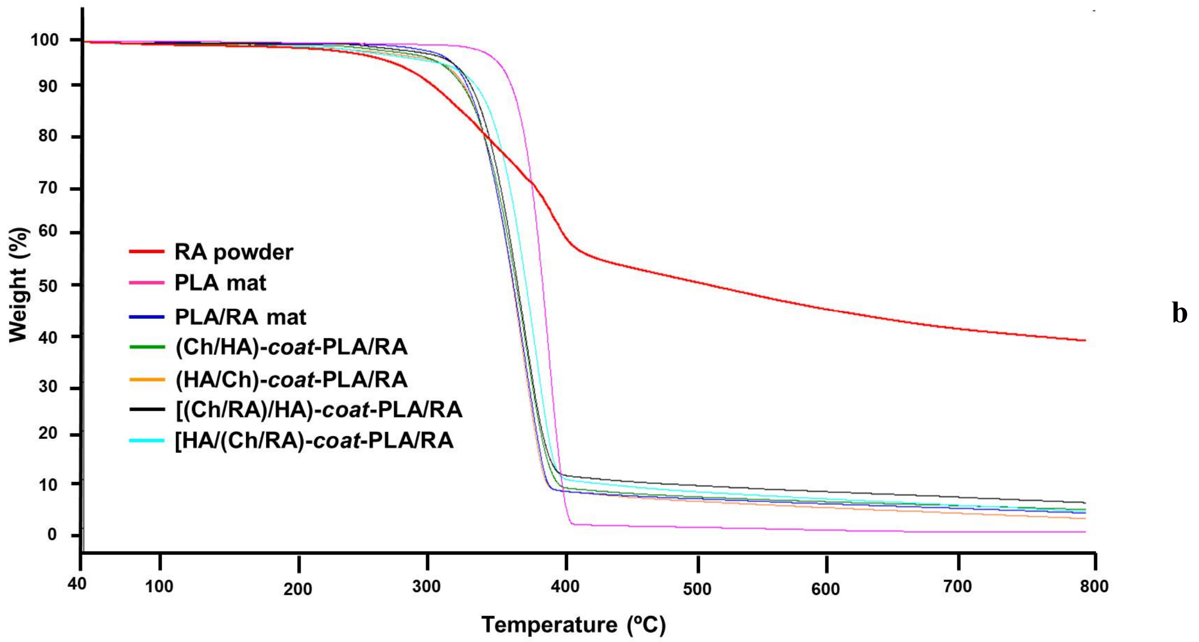

Figure S11. TGA thermograms of PEC-coated PLA (a) or PLA/RA (b) mats.

Table S3. Tensile characteristics of the fibrous materials

**Fibrous Materials**	**Tensile strength at Maximum Load [MPa]**	**Young's modulus [MPa]**	**Elongation at Break, %**
	**Electrospun PLA-based materials**		
PLA	3.48 ± 0.10	192.80 ± 6.71	20.39 ± 4.56
(Ch/HA)-*coat*-PLA	5.00 ± 0.01	350.99 ± 35.89	14.72 ± 1.30
[(Ch/RA)/HA]-*coat*-PLA	5.00 ± 0.39	361.49 ± 1.96	10.89 ± 1.96
(HA/Ch)-*coat*-PLA	4.50 ± 0.43	321.85 ± 31.68	17.56 ± 0.79
[HA/(Ch/RA)]-*coat*-PLA	5.09 ± 0.26	430.11 ± 17.71	16.78± 0.43
	**Electrospun PLA/RA-based materials**		
PLA/RA	3.77±0.14	192.52±2.97	23.67 ± 4.56
(Ch/HA)-*coat*-PLA/RA	4.24±0.03	190.45±7.39	21.78 ± 2.91
[(Ch/RA)/HA]-*coat*-PLA/RA	4.28±0.42	178.96±15.26	20.45 ± 0.45
(HA/Ch)-*coat*-PLA/RA	4.61±0.41	263.75±15.08	14.78 ± 0.96
[HA/(Ch/RA)]-*coat*-PLA/RA	4.38±0.35	207.46±16.10	23.11 ± 5.52

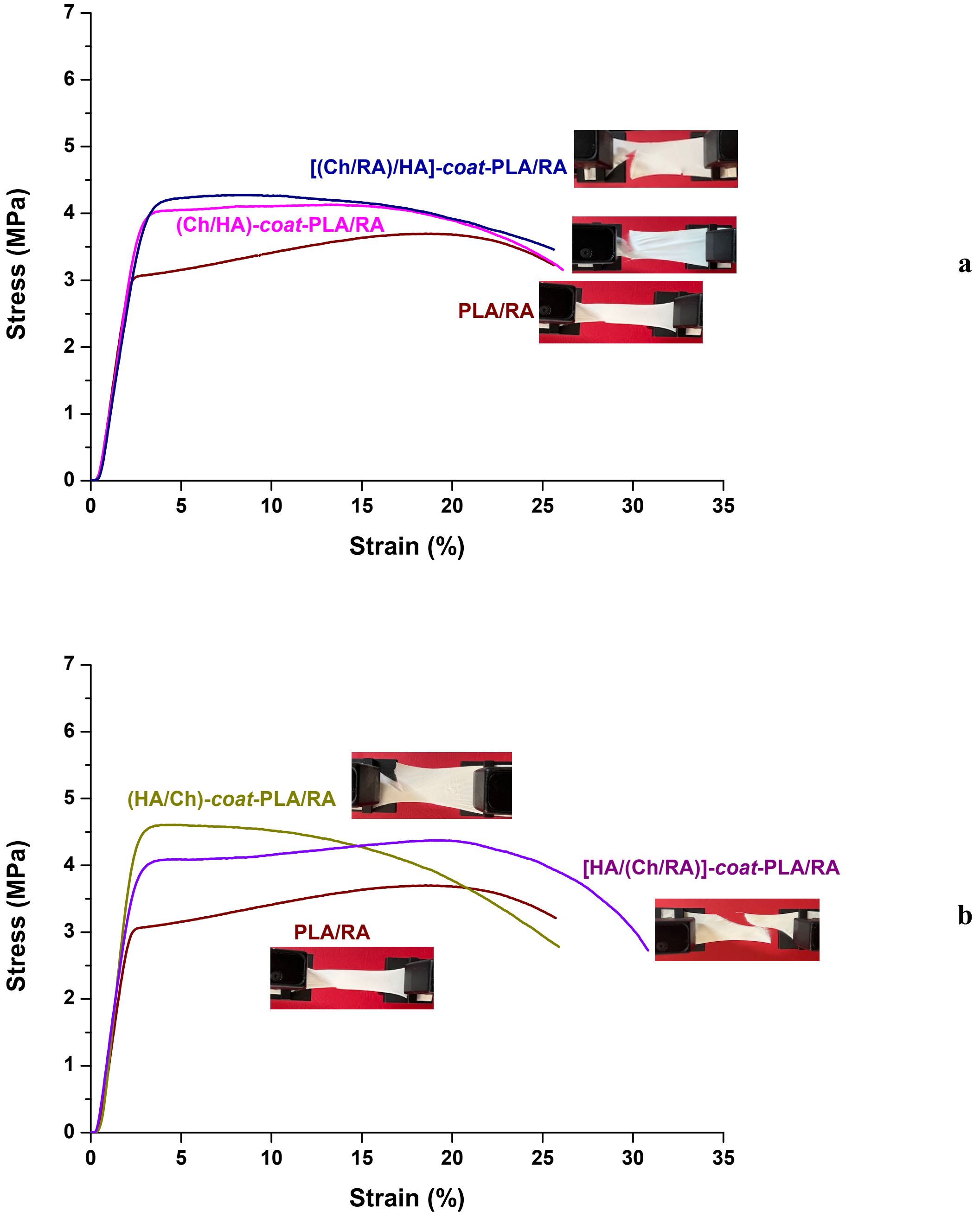

Figure S12. Stress-strain curves of PLA/RA-based fibrous materials.

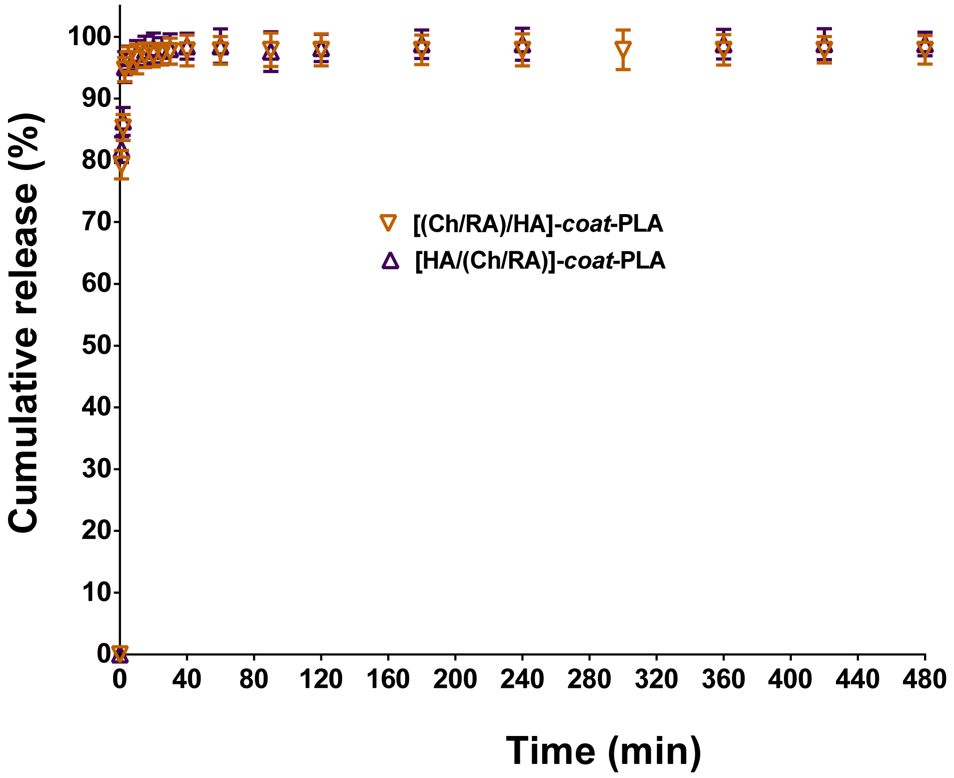

Figure S13. Release profiles of RA from mats: [(Ch/RA)/HA]-*coat*-PLA and [HA/(Ch/RA)]-*coat*-PLA in PBS (pH 7.4, I=0.1); 37 °C.

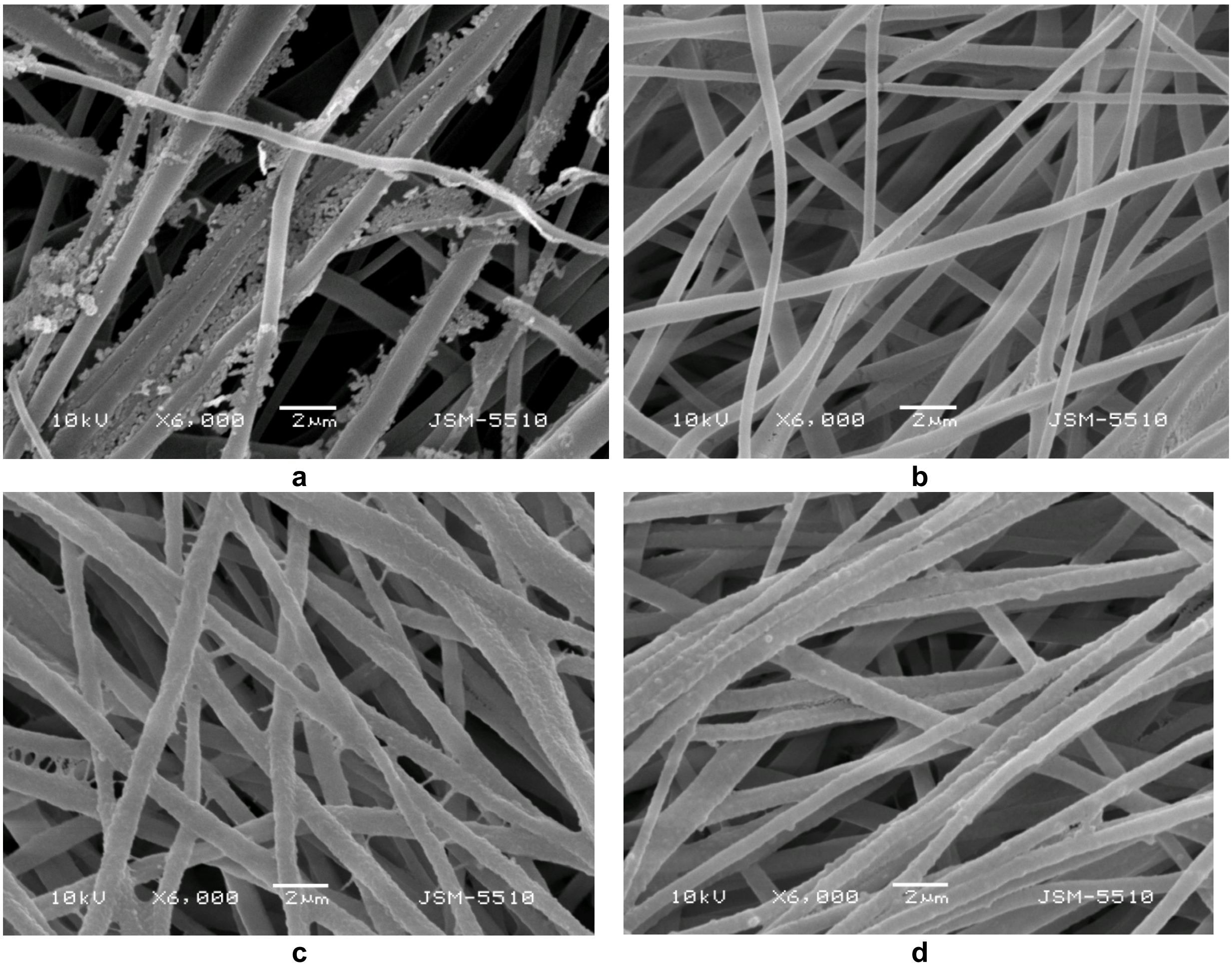

Figure S14. SEM micrographs of fibrous materials that have been incubated in *S. aureus* cell culture (10^7 cells/mL) for 24 h at 37°C, (a) PLA, (b) PLA/RA, (c) [HA/(Ch/RA)]-*coat*-PLA/RA and (d) [(Ch/RA)/HA]-*coat*-PLA/RA ; magnification ×6000.
